# Co-packaged optics (CPO): status, challenges, and solutions

**DOI:** 10.1007/s12200-022-00055-y

**Published:** 2023-03-20

**Authors:** Min Tan, Jiang Xu, Siyang Liu, Junbo Feng, Hua Zhang, Chaonan Yao, Shixi Chen, Hangyu Guo, Gengshi Han, Zhanhao Wen, Bao Chen, Yu He, Xuqiang Zheng, Da Ming, Yaowen Tu, Qiang Fu, Nan Qi, Dan Li, Li Geng, Song Wen, Fenghe Yang, Huimin He, Fengman Liu, Haiyun Xue, Yuhang Wang, Ciyuan Qiu, Guangcan Mi, Yanbo Li, Tianhai Chang, Mingche Lai, Luo Zhang, Qinfen Hao, Mengyuan Qin

**Affiliations:** 1 School of Optical and Electronic Information, Huazhong University of Science and Technology, Wuhan, 430074 China; 2 Wuhan National Laboratory for Optoelectronics, Huazhong University of Science and Technology, Wuhan, 430074 China; 3 Department of Electronic and Computer Engineering, The Hong Kong University of Science and Technology, Hong Kong, China; 4 HKUST Fok Ying Tung Research Institute, Guangzhou, 511462 China; 5 The Hong Kong University of Science and Technology (Guangzhou), Guangzhou, 511462 China; 6 Chongqing United Micro-Electronics Center (CUMEC), Chongqing, 401332 China; 7 Hisense Broadband Multimedia Technologies Co., Ltd., Qingdao, 266000 China; 8 Institute of Microelectronics, Chinese Academy of Sciences, Beijing, 100029 China; 9 State Key Laboratory of Superlattices and Microstructures, Institute of Semiconductors, Chinese Academy of Sciences, Beijing, 100083 China; 10 School of Microelectronics, Xi’an Jiaotong University, Xi’an, 710049 China; 11 Zhangjiang Laboratory, Shanghai, 201210 China; 12 The State Key Laboratory of Advanced Optical Communication Systems and Networks, Department of Electronic Engineering, Shanghai Jiao Tong University, Shanghai, 200240 China; 13 Huawei Technologies Co., Ltd., Shenzhen, 440307 China; 14 College of Computer, National University of Defense Technology, Changsha, 410073 China; 15 Institute of Computing Technology, Chinese Academy of Sciences, Beijing, 100086 China

**Keywords:** Co-packaged optics, Silicon photonics, High-performance computing, Advanced packaging, External laser, Optical power delivery, Co-simulation, Standardization, Transmitter, Receiver

## Abstract

Due to the rise of 5G, IoT, AI, and high-performance computing applications, datacenter traffic has grown at a compound annual growth rate of nearly 30%. Furthermore, nearly three-fourths of the datacenter traffic resides within datacenters. The conventional pluggable optics increases at a much slower rate than that of datacenter traffic. The gap between application requirements and the capability of conventional pluggable optics keeps increasing, a trend that is unsustainable. Co-packaged optics (CPO) is a disruptive approach to increasing the interconnecting bandwidth density and energy efficiency by dramatically shortening the electrical link length through advanced packaging and co-optimization of electronics and photonics. CPO is widely regarded as a promising solution for future datacenter interconnections, and silicon platform is the most promising platform for large-scale integration. Leading international companies (e.g., Intel, Broadcom and IBM) have heavily investigated in CPO technology, an inter-disciplinary research field that involves photonic devices, integrated circuits design, packaging, photonic device modeling, electronic-photonic co-simulation, applications, and standardization. This review aims to provide the readers a comprehensive overview of the state-of-the-art progress of CPO in silicon platform, identify the key challenges, and point out the potential solutions, hoping to encourage collaboration between different research fields to accelerate the development of CPO technology.

**Graphical Abstract:**

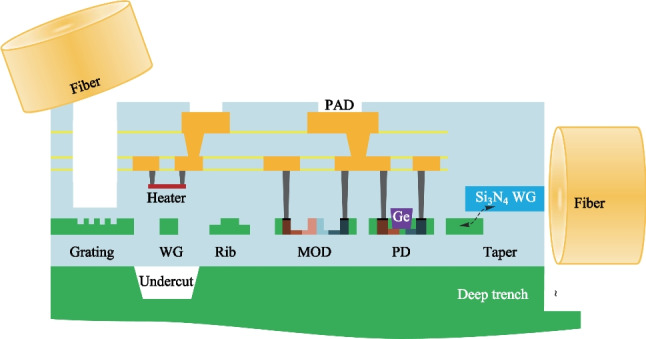

## Introduction^1^

*The importance of co-packaged optics* (*CPO*). Datacenter traffic keeps growing with the expansion of data-intensive applications, such as AI and high-performance computing (HPC). Conventional pluggable optics cannot catch up with the fast-growing bandwidth density and energy efficiency requirements. Co-packaged optics (CPO) combines photonic devices with high-performance electronics via advanced packaging to form a solution that shortens the SerDes distance significantly, greatly reducing power consumption.

*Aim and Organization*. The primary purpose of this paper is to provide an overview of the state-of-the-art progress of CPO, and identify the key challenges and their potential solutions. It is worth noting that the content in this paper is by no means exhaustive for such a rapidly developing field. To provide readers a comprehensive overview, we divide the paper into twelve independent sections. Here, we provide a brief overview of these sections.

*2. Device fabrication*. An advanced fabrication process and device structure need to be developed for CPO. In the form of 3D integrated CPO, the silicon photonic chip serves as an interposer for shorter traces and lower power consumption. In addition, standard silicon photonics fabrication technology must collaborate with the packaging development.

*3. External laser source*. The requirement for the laser chip is analyzed. It turns out that the high-power laser and TEC are the primary contributors. Potential solutions to reduce laser power consumption are proposed.

*4. Optical power delivery*. The optical power delivery system has often been oversimplified or even neglected in recent proposals. This section attempts to address the fundamental problems in optical power delivery from three aspects, specifically, how the power demands are growing, what technologies are required, and what the major challenges are.

*5. DSP for CPO*. The DSP chip plays an important role in CPO. This section summarizes the electrical requirements for both host-side and line-side links and provides the DSP design considerations, including the transceiver architecture, clocking scheme, and equalization implementations.

*6. Microring-based transmitter array for CPO*. Micro-ring modulator has small area, high power efficiency, and is compatible with wavelength division multiplexing, making it a promising candidate for CPO. However, it suffers from many challenges, such as wavelength control and polarization sensitivity. This section summarizes the challenges and recent advances of microring-based transceiver array and provides suggestions to meet these challenges.

*7. Mach–Zehnder modulator* (*MZM*) *based transmitter for CPO*. MZM has already been commercialized and is a promising solution to replacing the existing implementation of pluggable optical modules. However, MZM driver design poses numerous challenges in terms of voltage swing, bandwidth, energy efficiency, and other aspects. This section provides an overview of the MZM transmitter with a focus on its driver design.

*8. Optical receiver front-end for CPO.* Compared to BiCMOS, the CMOS-based optical receiver is more compatible with CPO in terms of integration, power efficiency, and cost. This section will provide recent advancements in the design of CMOS-based optical receiver front-end electronics, which hopefully will pave the way for future fully integrated electronics IC for CPO.

*9. 2.5D and 3D packaging for CPO*. 2.5D, 3D packaging technology could achieve high bandwidth and high integration with low power consumption for CPO. This section mainly discusses 2D/2.5D/3D silicon photonic co-packaging module developed by IMECAS, 2D MCM photonic module package issues, and the challenges of silicon photonic wafer-level packaging.

*10. Electronic-photonic co-simulation for CPO*. Electronic-photonic co-simulation is the prerequisite for large-scale electronic-photonic co-design. However, this field is relatively immature and faces many methodological and engineering challenges. The mainstream approach is to integrate the photonic devices into the electronic design automation platform. This section mainly discusses the challenges and solutions for photonic device modeling, time domain simulations, and frequency domain simulations.

*11. System considerations on HPC photonic interconnect*. This section breaks down the photonic interconnect link into hardware and software components, discusses accordingly their current status, challenges, as well as how they impact the integrity of the photonic link and network. Finally, this section remarks on the next milestone in the future of photonic interconnect for HPC networking.

*12. Optoelectronic hybrid interface in HPC.* HPC has been reluctant to switch to new technology due to compatibility considerations. So far, optoelectronic hybrid integration has failed to take advantage of the integration truly. This section analyzes different interconnecting design considerations for CPO and provides suggestions for accelerating the adaption of CPO in HPC.

*13. CPO development and standardization*. The China Computer Interconnect Technology Alliance (CCITA) has coordinated the efforts across academia and industry to initiate the China CPO standardization. This section provides an overview of the technical and economic considerations of the China CPO standardization efforts.

## Advanced silicon photonics fabrication technology for CPO^2^

### Status

Co-packaged Optics (CPO) is an advanced packaging technology for optoelectronic devices that involves upgrades in system architecture, chip fabrication, and packaging. In this section, we will mainly discuss the fabrication technology of silicon photonic chips for CPO applications.

Moore’s Law is a well-known phenomenon in microelectronics chip fabrication. During the last few decades, the number of transistors per chip has been doubling every two years. Similarly, silicon photonics, which claims to benefit from the existing and well-established Complementary Metal-Oxide Semiconductor (CMOS) manufacturing technology, should also follow this scaling trend and aim for the low-cost manufacturing of photonic integrated circuit (PIC) through the economies of scale [25]. However, unlike electronic devices, the scaling of photonic devices is intrinsically difficult. The size of a photonic device is mainly determined by the refractive-index contrast of materials. The global size of silicon photonic devices remains at the micrometer level and will rarely decrease to the nanometer level. Therefore, when we talk about the scaling of silicon photonics, we are talking about how advanced fabrication technology can enable the scaling of photonic packaging.

### Current and future challenges

**Fig. 1:**
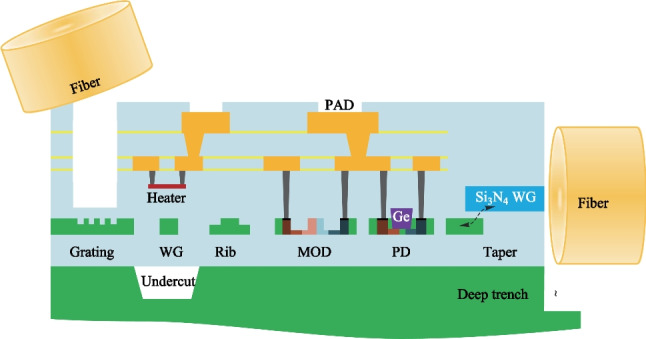
Schematic of CUMEC silicon photonics PDK

Pure-play foundries, such as TSMC, Global Foundry, TowerJazz, SMIC, and open-access pilot lines, such as IMEC, AMF, AIM, CUMEC are providing silicon photonics PDK with the basic component library of passive and active devices, as shown in Fig. 1. While customized structures are needed for CPO applications, the main fabrication challenges for CPO chips come from fiber coupling and light source integration.

**Fig. 2:**
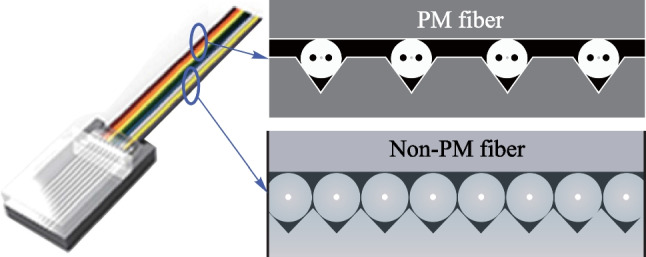
Hybrid packaging of PM fibers for light input and non-PM fibers for light output

An efficient fiber coupling structure is necessary for extreme high-density optical I/O. There are two kinds of coupling structures, grating coupler and edge coupler. Grating couplers are normally fabricated via a simple two-step etch process, enabling vertical light coupling. Grating couplers possess a relatively wide alignment tolerance with small optical bandwidth and high polarization sensitivity. Therefore, unlike edge couplers, grating couplers are usually used in wafer-scale testing instead of commercial products. Edge couplers enable small coupling loss and large optical bandwidth, which are desirable for real applications. However, edge coupler requires an undercut and deep etch process during the fabrication process, inducing problems in device stability and reliability. Besides, V-groove structure is developed for passive alignment of fiber edge coupling [26].

On-chip light source integration is one of the main challenges in silicon photonics. It is inherently difficult for silicon-based materials to form a high-performance laser. Heterogeneous integration or heterostructure integration of III-V compound materials on silicon photonic chips is proven to be a viable approach, while major adjustment is required for silicon photonic fabrication process.

In the future, from 2.5D CPO to 3D CPO, CPO technology will evolve to be more than a packaging process, but rather a combination of fabrication and packaging where the co-optimization of design and process is demanded. The packaging concept needs to merge deeply with fabrication process flow.

### Advances in science and technology to meet challenges

In most current CPO solutions, edge couplers are used in both light-in and light-out paths. The edge coupler is carefully designed to meet the demand of high alignment tolerance and low insertion loss simultaneously. Typical fiber-to-chip loss via passive alignment using V-groove structures could be controlled within − 1.5 dB [27]. Using structures such as thermal phase shifters could help further improve the alignment tolerance [28]. Silicon photonic transceiver serves as an important building block for high-speed switch assembly CPO system, in which several transceiver modules are arranged in close proximity to the switch ASIC. Central switch ASIC is surrounded by hundreds or thousands of fibers with a mixture of polarization-maintaining (PM) fiber and non-PM fiber, posing considerable challenges to fiber routing and packaging with high consistency and quality, as shown in Fig. 2. Adopting high order modulation technology and on-chip light source integration can reduce the number of fibers and difficulty of fiber packaging.

Furthermore, the wavelength-division multiplexing scheme or TeraPHY [29] could be another solution to addressing larger data flow.

**Fig. 3:**
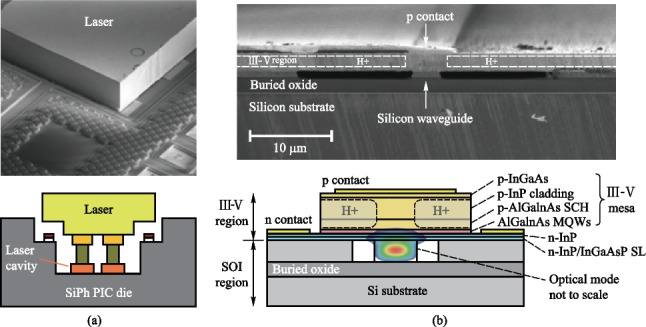
**a** Heterostructure integration [30] and **b** heterogenous integration [33] of on-chip light source

On-chip light source integration methods include heterostructure integration (e.g., laser diode flip-chip bonding) and heterogenous integration (e.g., wafer-level material bonding) (Fig. 3). For the flip-chip bonding method, commercial laser diodes are bonded via eutectic soldering on the silicon photonic chip. Mechanical stops and fiducial marks are used for high precision passive alignment between laser chip and silicon photonic chip [33]. This method utilizes the mature laser diode product for simplified development and quick commercialization. For the wafer-level material bonding method, lasers are formed together during the silicon photonic chip fabrication process [31–33]. The mode converter between III-V material and silicon waveguide requires process modification in the front-end of the line. Laser electrode fabrication induces process altering in the back-end of the line. Overall, the silicon photonic production line requires massive reconstruction for heterogenous integration. For both methods, heat dissipation and strain-induced performance degradation need to be considered for future applications in CPO.

**Fig. 4:**
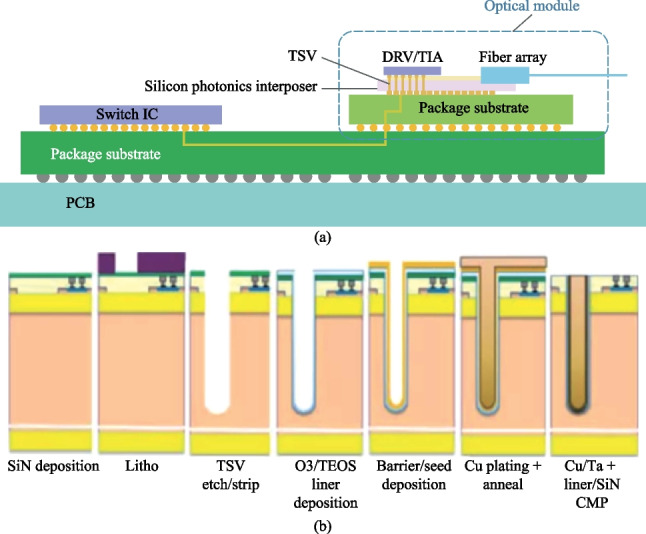
**a** Hybrid-assembled optical module using silicon photonic interposer with TSV structures [10]. **b** TSV fabrication process on silicon photonics interposer [35]

In the form of 3D integrated CPO, the silicon photonic chips serve as an interposer for shorter circuit connections and lower power consumption. Recently, imec has demonstrated a hybrid-assembled optical module embedded with through-silicon via (TSV) structure reaching above 110 GHz RF bandwidth, paving the way for next-generation silicon photonic modules operating at 100Gbaud data rates, as shown in Fig. 4 [34]. The fabrication of TSV on silicon photonic chips requires extra processes, including high-aspect-ratio Bosch deep etch and wafer thinning, which induces potential problems in yield and reliability [35].

### Concluding remarks

Following the trend of integration, standard silicon photonics fabrication technology must adapt with the development of packaging. In order to meet the requirements of CPO, advanced fabrication processes and device structures need to be developed for silicon photonics. It would be more efficient for CPO application designers to work closely with foundries for design-process co-optimization.

## External laser source for co-packaged optics^3^

### Status

The laser source is one of the enabling technologies for co-packaged optics (CPO). In the context of silicon photonics based optical engine, two types of laser source are under discussion and development, i.e., on-chip laser and external laser. Each approach has its pros and cons. This session focuses on the option of external laser source (ELS), mainly due to its wider accessibility to the industry.

**Fig. 5:**
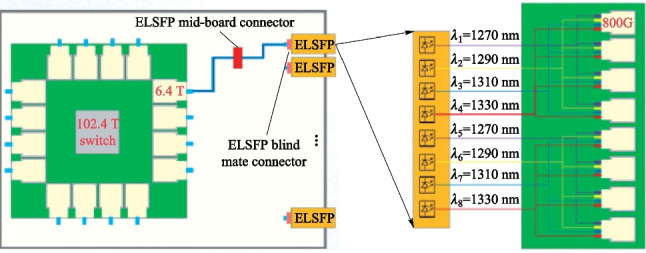
Configuration of co-packaged optics for 102.4 T

It is believed that the optical connectivity will more likely evolve to CPO form when the switching capacity reaches 102.4 Tbit/s for the data center networking (DCN) application. The CPO tile with a 6.4 Tbit/s optical input/output capacity is required by the 102.4 Tbit/s switch, as shown in Fig. 5.

The implement method of the 6.4 Tbit/s CPO tile is still under discussion, such as the data rate per lane and parallel or WDM architecture. For the sake of discussion, it is assumed that the 6.4 Tbit/s CPO tile is composed of eight sets of 800 Gbit/s cells, which are implemented by 4 × 200 Gbit/s FR4 configuration. Each 6.4 Tbit/s CPO tile requires an ELS. As shown in Fig. 5, each ELS package is composed of two sets of CWDM4 lasers, i.e., eight lasers in total. Each laser chip powers up to four 800 Gbit/s cells by utilizing a 1 × 4 splitter.

### Current and future challenges

The output power and power consumption are the key features of ELS. The output power requirement of ELS can be derived from the link budget analysis of optical engines.

Table 1 shows the link budget analysis for the output power. It is assumed that the minimum required output power (at TP2) of the optical engine is 0.2 dBm, according to the specification of 800G FR4 [72]. The total insertion loss of the silicon photonic chip is 21.6 dB, as shown in Table 1. Therefore, the minimum required output power of the ELS package is 21.8 dBm. The required output power of the laser chip is 24.5 dBm when taking the laser-to-fiber coupling loss into account and leaving room for margin.

**Table 1.**
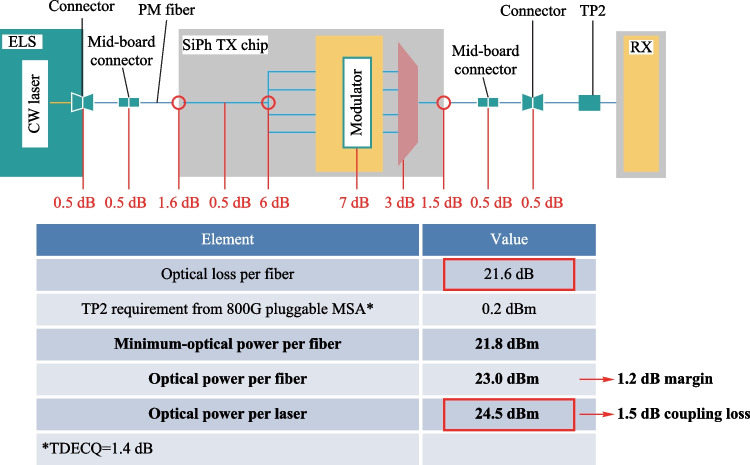
Link budget analysis for the optical engine

The power consumption of the ELS package is another critical parameter. Table 2 shows that the total power consumption of the ELS package for a 6.4 Tbit/s CPO is approximately 18 W. The laser chips and thermoelectric coolers account for nearly 70% of total power consumption.

**Table 2:**
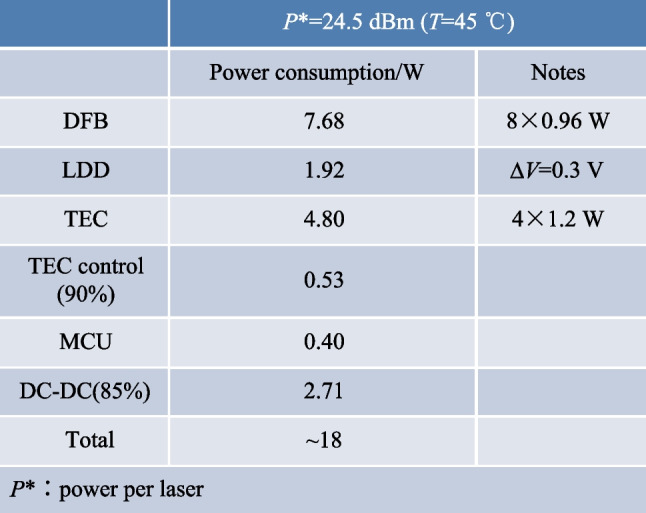
Total power consumption of ELS package

The high output power of the laser chip is the root cause for the majority of the power consumption. The wall-plug-efficiency of the laser chip is critical to power consumption and is defined as the ratio of optical output power to consumed electrical input power. The wall-plug-efficiency used in Table 2 is approximately 0.3, meaning that only 30% of electrical input power can be converted to optical output power, while the remaining power is dissipated as heat. Furthermore, the thermoelectric cooler (TEC) consumes additional electrical power to dissipate the heat generated by the laser chip. The total power consumption of 16 ELSs, which are required for a 102.4 Tbit/s switch, is 288 W.

The form factor of ELS is being standardized in OIF [73], including the electrical and optical interface, footprint, management interface, etc.

### Advances in science and technology to meet challenges

**Fig. 6:**
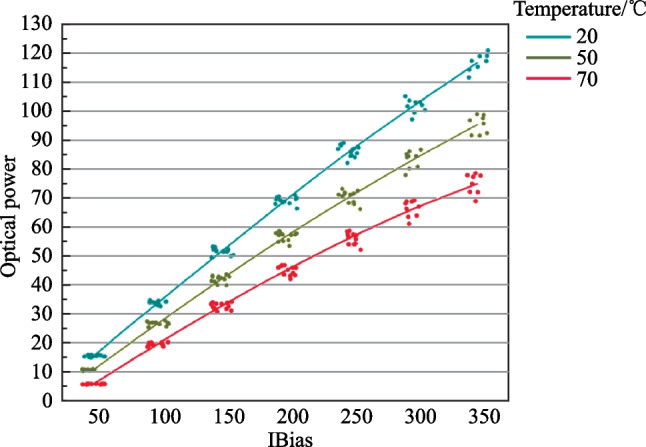
Performance of high-power laser

*1. High output power*. The existing CW lasers developed for pluggable transceivers cannot meet the high output power requirement of CPO applications. The silicon photonics based pluggable optical transceivers, e.g., 400G DR4, generally require CW lasers with < 100 mW output power. In contrast, CPO applications require much higher output power for the CW laser. As shown in Table 1, the required output power of the laser chip is 286 mW for the WDM architecture. Although the output power is much lower for the DR architecture, at least 100 mW is still required. For industrial applications, a slab-coupled optical waveguide DFB laser diode at C/C + bands with a kink-free CW output power exceeding 100 mW has been reported [74]. However, at O band, we have developed a 1310 nm CW laser that can only reach 80 mW at 50°C, as shown in Fig. 6. CWDM4 lasers with only about 70 mW output power have been reported [75]. Therefore, there is a need to develop high-power lasers for CPO applications.

*2. High wall-plug-efficiency*. In addition to high output power, high wall-plug-efficiency is another desirable feature of the high-power CW laser from the energy efficiency point of view. Moreover, a thermally efficient TEC is helpful for the reduction of ELS power consumption. Furthermore, an uncooled high-power laser might be the ultimate solution to the CPO light source.

*3. Monolithic integration.* Silicon photonic platform processes are well established and allow for higher overall transmission and reception performance than CMOS and BiCMOS platform, but laser integration remains a challenge for all silicon platforms and subject of active research. The previous major challenges of monolithic integrating III-V lasers on Si platform have been the impaired device performance due to materials dissimilarity. Recently, III-V QD lasers monolithically grown on Si substrates have demonstrated very promising results with a long lifetime, high output power, and low threshold current densities. However, in order to realize the application leveraging the monolithic integration of QD lasers on SOI platform, optical coupling to waveguides must be resolved. In contrast, InP based platform can readily integrate active materials, rely on a stronger electro-optic (EO) Kerr and Pockels effect, and achieve higher EO bandwidth-efficiency metrics. Further enhancement of the EO effect can be accomplished with the quantum-confined Stark effect in quantum wells, but at the cost of higher temperature and wavelength dependence.

### Concluding remark

External laser source is a promising solution to the CPO light source due to its easy maintenance and wide accessibility. Standardization of ELS in OIF is underway and will accelerate the maturity of the technology. High power CW lasers with at least 100mW output power are desired to meet the link budget requirement. The wall-plug-efficiency of CW lasers requires further improvement for power-saving purposes. Monolithic integration of external laser source benefits from smaller parasitic capacitances and lower packaging cost, making them the most promising solution for achieving reliable, power efficient, high-density integration of laser diodes on silicon chips.

## Optical power delivery in multiprocessor systems^4^

### Status

With the maturity of nanophotonics, chip-scale optical networks are projected to be the enabling technology to sustain the continued performance scaling of future multiprocessor systems. This is because optical links are capable of providing intra-chip (e.g., core-to-cache) and inter-chip (e.g., core-to-memory) communication with orders-of-magnitude higher signal fidelity, lower latency, and higher bandwidth density via wavelength division multiplexing (WDM) compared to traditional electronic links [217]. Moreover, the unique properties make a chip-scale optical network more than just a simple replacement to its electrical counterpart, but rather a brand-new architectural inspiration.

Since the first proposal of Goodman et al. [218] in 1984, optical interconnection networks have become promising to offer high-quality transmission in applications ranging from high-performance computing [219], memory access networks [220, 221], data center networks [222], to even analog computing [223]. The continued progress makes this technology a natural solution to the fundamental conflicts between performance scaling and thermal constraints in future multiprocessor systems.

While remarkable, these pioneering designs are often hard to scale due to the proliferation of laser sources whose power and packaging cost can more than negate the abovementioned benefits. For a 256-core system, Corona [220] consumes as much as 58.51 pJ/bit power at best [224], I2CON requests an integration of up to 4096 lasers [225], PROBE would require at least 64 fibers attached to a single chip for power provisioning only [226]. While these numbers are still barely feasible, scaling up the system either leads to high packaging cost, complicates layout, or is entirely impractical.

The optical power distribution networks (OPDNs), on which laser light is distributed to its modulation phase, play a critical role in the system power consumption [227]. Despite the importance, floorplanning, as well as placement and routing (P&R) are less discussed in the literature because the underlying technologies are still admittedly in their early stages. Nonetheless, significant industrial and academic efforts have centered around the silicon photonic platform thanks to its compatibility with the bulk CMOS process flow and mature infrastructure.

The trend is further boosted by a combination of the cost-sharing business model and commercial availability through international foundries, such as Global Foundries, Freescale, TSMC, IMEC, CEA Leti, and IME. Therefore, as an early attempt, optical power distribution networks and their floorplan optimizations have mostly addressed silicon photonic platforms in the past few years [227, 228].

### Current and future challenges

**Fig. 7:**
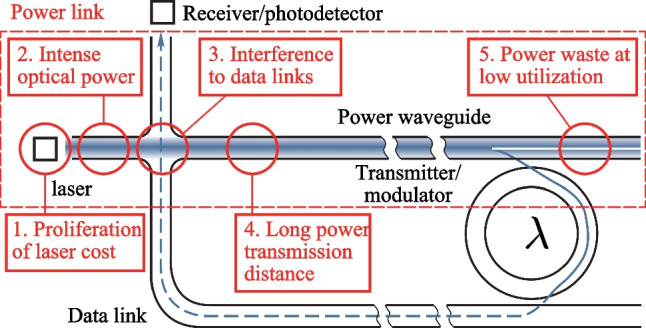
Challenges of optical power delivery illustrated by a simple optical link

Despite the recent developments, the design paradigm for supplying power to the compact yet dense chip-level optical links has not been established thus far and several technological obstacles must be addressed before massive adoption in commercial multicore architectures is possible. Laser power consumption contributes to over 70% of the network power [229] and has become one of the primary design constraints under the increasing demand for network capacity. To enable efficient optical power delivery, five main challenges are illustrated in Fig. 7.

*1. Proliferation of laser cost.* Hundreds to thousands of wavelength channels are making their way into chip-scale in recent works [219] as a result of the increasing demand on optical networks. However, the low energy conversion efficiency of O- and C-band lasers, ∼10% at best [230], and the lack of efficient packaging technologies make it hard to integrate laser sources at a large scale. While the debate over the use of off- v.s. on-chip laser sources is ongoing, a highly efficient, easy-to-integrate laser source would surely be favorable.

*2. Intense optical power-induced power loss.* A silicon waveguide can become opaque when exposed to high optical power despite being known for its transparency in the infrared spectrum. An extra 3 dB/cm loss is observed in a standard 250 nm × 450 nm strip waveguide with 50 mW of power injected [231] and the loss is projected to aggravate exponentially with increasing injected power. Theoretical explanations are given by modeling the two-photon absorption (TPA) and free-carrier absorption (FCA) phenomena in waveguides [232]. To overcome the fundamental power capacity constraint of waveguides, it would require a cross-layer effort, from conscious architecture designs and better floorplanning, to advanced material platforms.

*3. Interference to data links.* As an optical signal propagates through data links, it is attenuated by multiple factors, such as waveguide micro-resonator loss and crossing reflection, causing increased network loss and noise. An OPDN exaggerates the attenuation by introducing an extra amount of waveguide crossings after the P&R phase. A recent study on a 16 × 16 network has reported a 1 × more laser power consumption and − 0.73 dB SNR at worst by considering the routing of the power distribution network [227]. Note here that the numerical results are subject to complex design choices in, for example, topology and P&R algorithm. Still, most recently published works, such as [233] and [234], have admitted that increased wiring intricacy largely aggravates the power loss and crosstalk noise. However, optimizing the data network and OPDN together regarding topology, floorplanning, and P&R is still challenging and requires comprehensive studies.

*4. Long power transmission distance.* Power transmission arises when a laser source is spatially shared among modulators. This is often the case because assigning one dedicated laser source to every modulator would either be expensive or totally infeasible. Ref. [227] evaluates 13 networks and reports an average of 16.71% power loss during transmission. Ref. [228] further points out that a better laser sharing scheme depends on the logical topology and the physical layout of the OPDN. However, there lacks an efficient design approach that optimizes the sharing and placement of lasers in the literature.

*5. Power waste during low link utilization.* A majority of power is wasted in an underutilized link when the lasers are kept on. This is not uncommon for many parallel applications where the network utilization is very low (less than 5%) with only a few traffic spikes [235]. Strategically turning off underutilized links or sharing lasers among links can mitigate the waste [228].

### Advances in science and technology to meet challenges

*1. Device and Circuitry.* Epitaxially grown quantum dot lasers are promising to attain low-power consumption and athermal performance on silicon photonic platforms, demonstrating the lowest threshold current and highest lasing temperature compared to alternatives [236]. Efforts to mitigate the nonlinear power loss in waveguides include a special p-i-n structured waveguide that cuts the carrier lifetime from 16 to 6.8 ns passively, and to 1 ns at 25 V reserve bias [237]. Additionally, an integrated grating and 16-way star coupler is capable of eliminating the high-intensity regions in waveguides and couples up to 275 mW optical power with only 0.45 dB additional loss [231]. Development on low-loss, low-crosstalk waveguide crossing has achieved ~ 0.007 dB loss and <  − 40 dB crosstalk [238].

*2. Topology of the power distribution network.* Proposals of power distribution networks can be classified into two types, namely static OPDNs and re-configurable OPDNs. Many pioneering designs adopted static OPDNs for their simplicity in fabrication and optimization, including bus-type [220], star-type [231], and binary tree [227] structures. Static OPDNs are cheap, easy-to-reason, but less responsive to fluctuation in power demands. Recent proposals tend to apply re-configurable OPDNs, which allow flexible power sharing between links in need [229, 235].

*3. Adaptive laser allocation.* Another way to save laser power is by avoiding over-provisioning via offline optimization or online adaptation. Researchers have found the former effective when applied to specific applications with regular communication patterns. An average of 74% power saving is achieved over a range of applications [239]. The adaptive power allocation scheme always presents a trade-off among network re-configurability, re-configuration overhead, and power saving. A laser power saving of 68% is demonstrated in Ref. [226], but at the cost of a 12% loss in performance.

*4. Advanced fabrication technologies.* Significant industrial and academic efforts have centered around silicon photonics with a preference on materials, including crystalline silicon, polycrystalline silicon, and silicon nitride. Apart from the conventional single-layer crystalline silicon, polycrystalline silicon or silicon nitride can be deposited to form additional layers above. The stacked structure benefits from the low-loss materials and eases P&R compared to its 2D counterpart [235].

### Concluding remarks

To meet the demands from the next-generation chip-scale optical networks, future optical power sources must aim to work with minimal lasers and power consumption while meeting the device and layout constraints. As a cross-layer effort, static approaches involve the advancement in circuitry, such as better materials, devices, floorplanning, and P&R, while dynamic approaches include runtime laser assignment and on-demand power allocation.

**Acknowledgements**

This work was partially supported by ACCESS and Foshan-HKUST Projects Program (FSUST20-FYTRI12F).

## DSP design considerations for co-packaged optics^5^

### Status

**Fig. 8:**
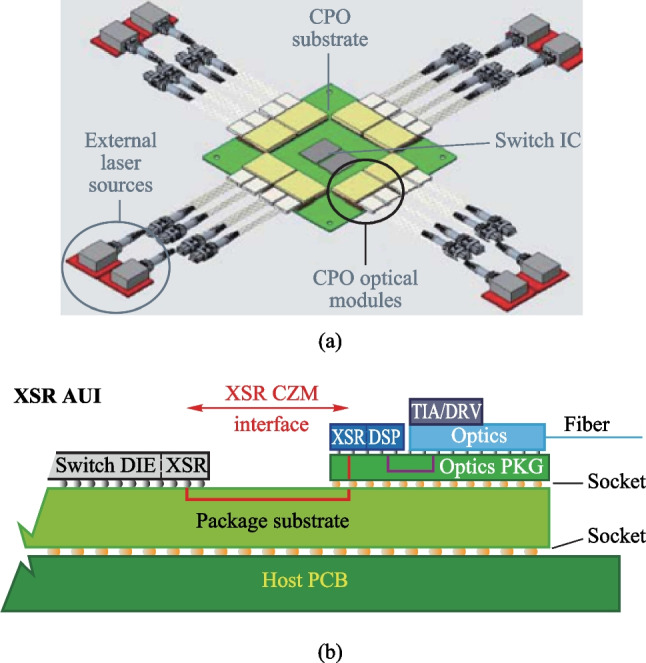
**a** Pluggable optical module [13]. **b** Architecture of CPO [13]

As data rate increases, traditional pluggable optical modules occupy a large volume, limiting the signal density to a certain extent (Fig. 8(a)) [13]. Figure 8(b) shows an architecture of co-packaged optics (CPO), where the optical engine and switching chip can be packaged together, the host-side of the re-timing chip is connected to the payload ASIC by XSR SerDes, and the line-side of the re-timing chip is connected to the optical engine by LR SerDes [13]. Thus, CPO effectively reduces power consumption, increases signal density, and reduces latency. This paper will focus on the design considerations of SerDes in CPO. Regarding current and future challenges, the architecture of the SerDes transceiver, as well as the requirements of SerDes on the host-side and the link-side are introduced. Then, we describe the advances in science and technology to meet challenges, including bandwidth, clock, and equalization.

### Current and future challenges

Although CPO demonstrates high performance in areas such as transmission data rate, there are also some challenges. In this section, we describe the requirements and challenges from two aspects: Host-side XSR SerDes and Line-side LR Serdes. The block digram of each SerDes is also provided.

*1. Host-Side XSR SerDes design consideration*

**Fig. 9:**
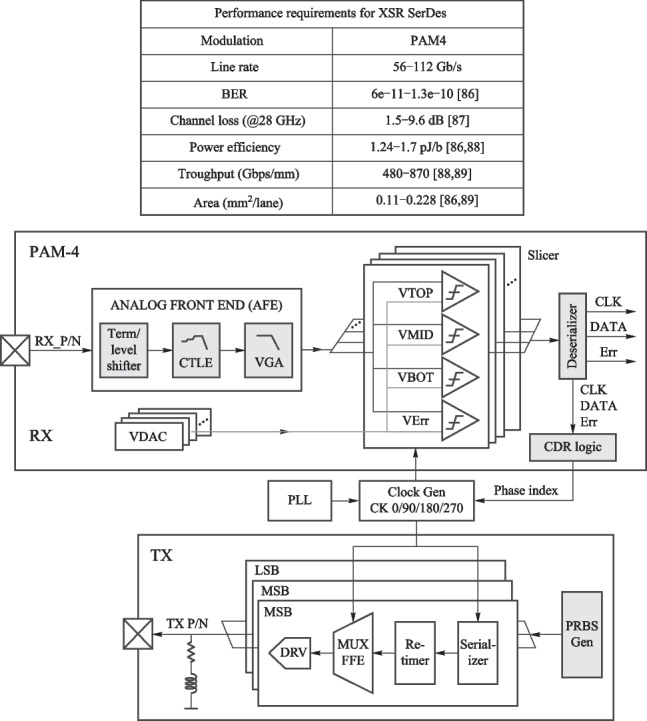
XSR top-level structure block diagram and XSR applications and requirements

The XSR SerDes is targeted to optimize power efficiency, integration density, and transmission latency by reducing the connection distance between two communication chips. Figure 9 summarizes the performance requirements for 56–112 Gb/s PAM4 XSR SerDes and conceptually shows the architecture of the XSR transceiver. The transmitter usually adopts an analog-mixed architecture or a 5 bit DAC topology with a CMOS MUX, several tap FFE, and a SST driver architecture to implement data serialization, wave pre-distortion, and output driving. The receiver often employs a simple continuous-time linear equalizer (CTLE) followed by a VGA and several slicers to directly extract the originally transmitted data. Similar to conventional transceivers, the XSR SerDes also needs a common PLL and a local CDR to adaptively track the optimal sampling points. Overall, the main feature of the XSR SerDes is utilizing a simple RX-side CTLE combined with a TX-side FFE to achieve high power efficiency and low code error rate, while handling a relatively low channel loss.

*2. Line-side LR SerDes design consideration*

The Line-Side LR SerDes is targeted to deal with non-ideal factors, such as finite bandwidth, chirp effect, noise, dispersion effect, and the nonlinearity of the device. Therefore, the equalization part of LR is more complicated compared to XSR, so the balance between bit error rate and energy consumption ratio should be ensured when designing.

**Fig. 10:**
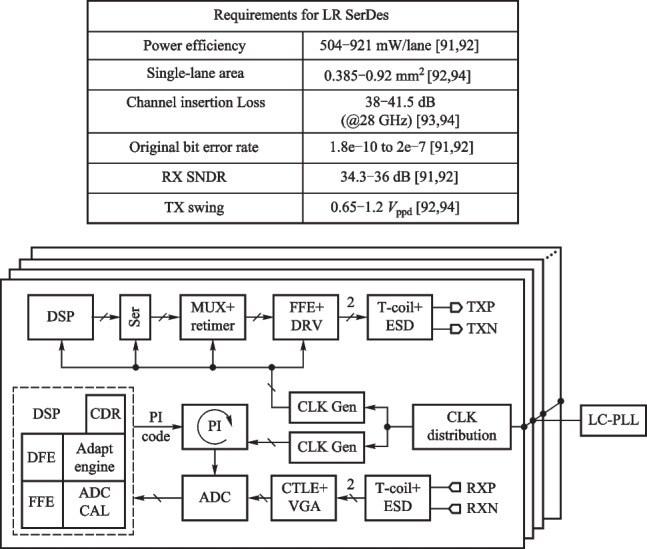
LR architecture and performance requirements

Figure 10 shows the LR architecture and performance requirements. The transmitter is usually a DSP composed of an FIR filter, MUX, multi-tap FFE, and DAC driver. DAC driver has SST form and CML form. The receiver is mainly composed of CTLE, VGA, ADC, and a DSP with calibration, equalization, and clock recovery. A common PLL and local CDR are used to determine the best sampling point for tracking.

### Advances in science and technology to meet challenges

From the system perspective, we provide notable advances in clocking scheme, bandwidth extension techniques, and equalization techniques.

*1. Clocking scheme*

**Fig. 11:**
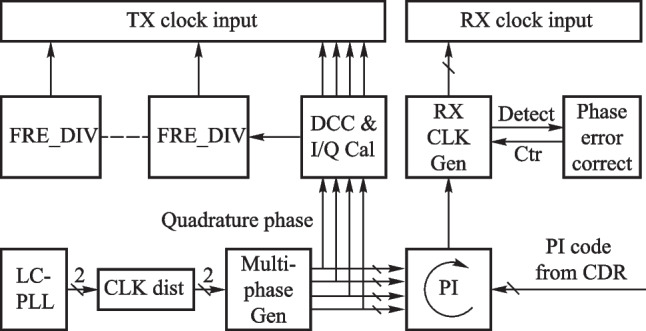
Clock path

Figure 11 shows a widely used clock scheme of wireline transceivers. The clocking scheme of mainstreamed and advanced transceivers mainly consists of LC-PLL, phase interpolator (PI), multi-phase clock generation, clock and data recovery (CDR), clock distribution, and clock adjustment modules [86, 88, 90, 94].

LC-PLL generates a high-frequency and adequately pure reference clock that acts as a metronome to control the transmitter process and serialize the data. By interleaved sampling in time with the multi-phase clock and high order modulation, the low-frequency clock can be used to accomplish the transmission of the high-speed bitstream. Multi-phase generation modules are generally implemented by Ring ILO (injection-locked oscillator) [95]. Due to the delay mismatch between the injection stage and other stages in the Ring ILO [94], as well as the non-ideal factors of the clock generation and transmission, the timing of the multi-phase clock will have a lot of jitter, and additional phase error correction loop is necessary to sense the error caused by the above factors and adjust the phase [94]. RX needs to sample the input data at the appropriate moment. CDR can provide phase information of input data and track its long-term jitter.

*2. Bandwidth extension techniques*

**Fig. 12:**
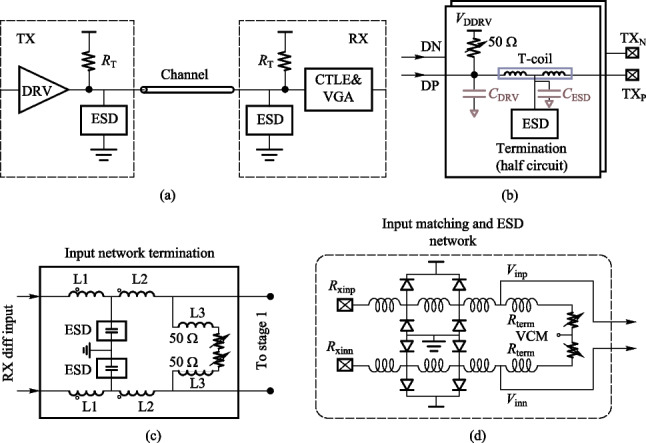
**a** Terminal of the transceiver. **b** TX with T-coil Peaking [97]. **c** RX with T_coil and inductive peaking [99]. **d** RX with LC-π network [100]

The large parasitic capacitance of the ESD device and PAD shown in Fig. 12(a) limits the bandwidth of the circuit and also causes a large return loss. Therefore, bandwidth extension and impedance matching methods are required.

Commonly used bandwidth extension methods include inductive peaking, T_coil peaking, and LC-π peaking. Compared to the 1.6 − 1.7 times bandwidth extension of the inductor peaking, the symmetric T_coil peaking can extend the bandwidth to 2.7 − 2.8 times [96]. Besides, T_coil is very suitable to combine with ESD to achieve high bandwidth, adequate electrostatic protection, and good impedance matching. Figure 12(b) shows a 128 Gb/s transmitter using T-coil to eliminate the ESD parasitic capacitance and achieve good impedance matching [97]. Note that sometimes asymmetric T_coil circuit may have better performance but no analytical expression, thus needed to be simulated and designed by electromagnetic simulation tools [98]. Figure 12(c) shows a 56 Gb/s PAM4 receiver that uses a combination of T_coil peaking and inductive peaking [99]. Figure 12(d) shows an LC-π network using 4-segment inductors to separate the capacitors, which achieves 56 GHz bandwidth and good impedance matching [100].

*3. Equalization techniques*

**Fig. 13:**
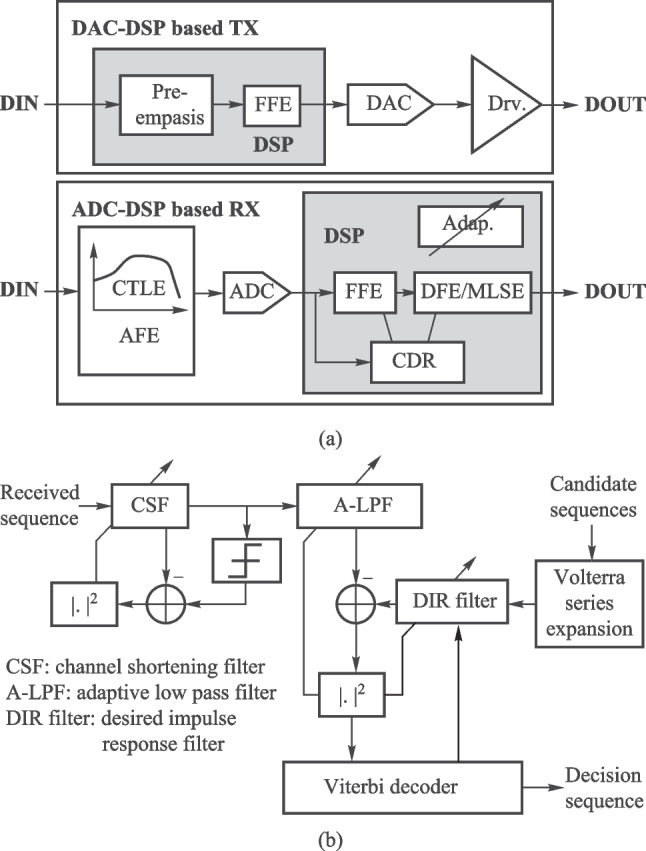
**a** Equalizers in ADC-DSP based SerDes. **b** DF-NL-MLSE [104]

Equalization is a critical technology that reduces ISI and improves the reliability of the entire communication system. In high-speed SerDes, selecting a specific equalization scheme is closely related to the characteristics of channel features and signal loss For example, FFE and DFE with more taps can be used with SerDes in LR with a poor channel environment (Fig. 13(a)) [90, 93]. However, the equalizer in XSR is simpler than that of LR due to the short length of the XSR interconnection channel built in CPO [86, 87, 101].

For digital FFE and DFE equalizers, the tap coefficient is directly related to the quality of equalization. Currently, the improved SS-LMS algorithm reduces the computational complexity and is widely used in equalizers. Other methods, such as PD-DLR [102] or machine learning based algorithm [103], can achieve a better adaptive result compared to the SS-LMS algorithm. However, when facing serious ISI circumstances, the performance of FFE and DFE equalizers will be degraded due to noise enhancement and error propagation. Although maximum likelihood sequence estimation (MLSE) equalization is rarely used in SerDes of CPO, the improved MLSE algorithm is still of certain reference for the design of nonlinear equalizer in high-speed receiver DSP. Figure 13(b) shows the MLSE equalizer architecture in the DSP [104].

### Concluding remark

DSP plays an important role in CPO transceiver. Compared to the hybrid architecture transceiver, many algorithms that can improve the performance of the entire system can be implemented in DSP based on CMOS technology. Although certain challenges remain in the way of achieving high performance and low power comsumption, many positive attempts to solving these problems from different aspects are given in this paper. The DSPs in CPO will always advance with the progress of semiconductor technology and design methodology, including clock scheme, bandwidth extention techniques, and high-efficiency equalization algorithms.

**Acknowledgements**

This work was partially supported by the National Natural Science Foundation of China (Grant No. 62074162).

## Microring-based transmitter array for co-packaged optics^6^

### Status

**Fig. 14:**
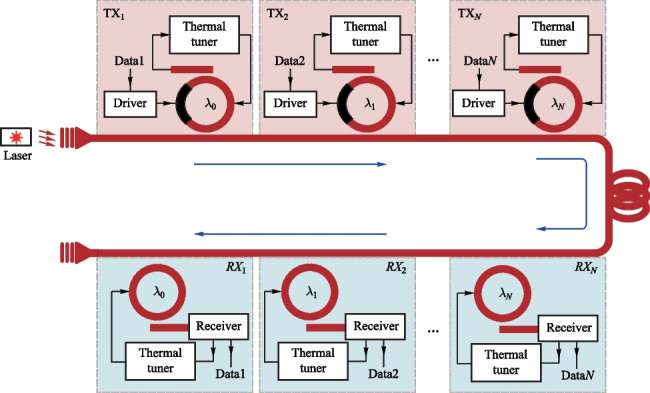
The diagram of MRR-based transceiver consisting of drivers, MRMs, receivers, MRR DEMUX, and thermal tuners

Microring (MRR)-based transceiver array (Fig. 14) is a promising solution to meeting the stringent bandwidth and power consumption requirements of future data centers due to their small size, high energy efficiency, and WDM-compatibility.

The high-bandwidth, low-power MRR-based transceiver array has already been demonstrated in hybrid integration [105] and monolithic integration platforms [106, 107]. In hybrid integration, the connection between the photonic chip and the electronic chip results in parasitics that limit the bandwidth and power consumption, such as the parasitic inductance (250 − 500 pH) and parasitic capacitance (~ 100 fF) caused by wire-bonding [108], as well as the parasitic capacitance (~ 30 fF) caused by micro-bump/copper pillar connection. However, 3D integration of electronic and photonic wafers performs with very low inter-connection parasitics capacitance (~ 3 fF) by through-oxide vias (TOVs) [109]. Monolithic integration reduces the parasitics of the wiring, improving the performance and increasing manufacturing costs. At the transmitting end, the data rate of a single micro-ring modulator (MRM) has reached 200 Gb/s [110]. At the receiving end, the data rate of a microring photodetector (MRPD) has reached 112 Gb/s [111]. However, laser integration compatible with mass-producible silicon technology is still an outstanding challenge [112].

Despite the integration challenges, the microring-based transceiver array has made rapid progress in recent years. Using hybrid integration, the single MRM-based transmitter has reached a data rate up to 112 Gb/s with 6-pJ/bit energy efficiency [113]. The MRM-based transmitter array has achieved a data rate up to 4 × 112 Gb/s with 5.8-pJ/bit energy efficiency [114]. Using monolithic integration, the single MRM-based transmitter has a data rate of 100 Gb/s with 0.7-pJ/bit energy efficiency [106]. The MRM-based transmitter array has shown a data rate up to 16 × 5 Gb/s with ~ 0.8-pJ/bit energy efficiency. In all these works, to stabilize the resonant wavelength of the MRM, closed-loop thermal tuning is required to compensate for the process variations, thermal fluctuations, and input laser wavelength/power variations [115, 116]. Using hybrid integration, a receiver based on CMOS technology has reached a data rate of 100 Gb/s with − 11.1 dBm sensitivity and 3.9 pJ/bit [117]. Using monolithic integration, the split-MRR resonant photodetector has a data rate of 12 Gb/s with 0.58 pJ/bit energy efficiency based on CMOS technology [118], and a 56 Gb/s optical receiver with 3.66 pJ/bit energy efficiency is implemented based on BiCMOS technology [119]. Closed-loop thermal tuning for MRR-filter is also necessary for wavelength locking of MRR-based receivers. In error-free transmission (bit error rate (BER) < 1e − 12), MRR-based transceiver array has reached 4 × 50 Gb/s in hybrid integration method [1] and 8 × 16 Gb/s with 4.96 pJ/bit energy efficiency in monolithic integration method [3].

### Current and future challenges

Several technological challenges remain to be tackled to further improve the performance and robustness of the MRR-based transceiver array. We outline these challenges below:

*1. Wavelength locking.* Due to the resonant wavelength characteristics, the performance of MRRs is susceptible to fabrication variations, thermal fluctuations, and input laser wavelength/power variations. If these non-ideal factors are not compensated, the work condition of MRR will not be stable, which will significantly degrade the modulation and DEMUX performance. Therefore, it is necessary to stabilize the resonant wavelength of the MRR in a variety of different environments. The closed-loop feedback control loop [113, 115, 116, 120–126] is often used to lock the resonant wavelength of the MRR, MRPD, and MRM. However, there is almost no work that can simultaneously compensate for fabrication variations, thermal fluctuations, input laser wavelength/power variations, and random data patterns.

*2. Modulation linearity.* Pulse amplitude modulation with 4-level (PAM-4) is commonly used to increase the data rate but puts forward new requirements on the linearity of the modulators. The modulation quality of PAM-4 is determined by the ratio of level mismatch (RLM), and we hope that the eye pattern distribution of PAM-4 is uniform [127]. However, due to the Lorentzian shape of the transmission curve, the MRM has static nonlinearity, making a uniform level of electrical PAM-4 eye pattern input to generate a non-uniform optical eye pattern output. Furthermore, the MRM PN junction capacitance is different at different bias voltages, resulting in different bandwidths at different voltage levels, introducing different inter-symbol crosstalk. This phenomenon is called dynamic nonlinearity [113]. How to compensate for the static and dynamic nonlinear effect of the MRM is a key factor in optimizing the performance of the MRM.

*3. Polarization handling.* For silicon photonic wavelength division multiplexing (WDM) receivers, the performance of the channel filter changes with respect to the polarization of the optical input from a single-mode fiber (SMF). Polarization-maintaining fiber (PMF) is used to maintain the polarization state in SMF, but it is too expensive to be applied in commercial applications [128]. How to control the polarization state effectively on-chip is the key challenge to reduce cost and improve performance.

*4. Circuit-level photonic-electronic co-simulation.* MRR-based transceiver array is a photonic-electronic integrated circuit [108]. Photonic device design is mainly a device-level simulation implemented by PDA tools [129], and electronic circuits are circuit-level simulations implemented by mature EDA tools. Device-level simulation can provide very accurate simulation results but cannot be directly applied to circuit-level simulation design due to its low simulation efficiency. How to perform circuit-level photonic-electronic co-simulation is a prerequisite for MRR-based transceiver array design. Thermal tuning for MRM and MRR is closed-loop electrical-optical-thermal process, and there is a big frequency mismatch between thermal fluctuation (~ kHz), electrical modulation (~ GHz), and light (~ THz), which will significantly increase the simulation time and reduce simulation efficiency.

### Advances in science and technology to meet challenges

Recent advances in photonic integration have addressed some of the challenges discussed above.

*1. Closed-loop thermal tuning.* To implement effective closed-loop thermal tuning for MRR, the relative position of the resonant wavelength and laser wavelength must be obtained using a monitor, such as photodiode [115] and contactless integrated photonic probe (CLIPP) [130]. Secondly, a controller is required to generate the control signal to align the resonance wavelength according to a suitable algorithm [115, 116, 121–124, 131] or analog control circuit [120]. To increase the tuning temperature range, a driver is required to provide sufficient current to heat the integrated resistor, thereby tuning the resonance wavelength through the thermo-optic effect [113, 132]. Unlike MRR, wavelength locking for MRM faces additional challenges. Optical bistability and data-dependent self-heating [133] will significantly reduce the performance of MRM. The average power detection based wavelength locking cannot perform optimally when the non-DC balanced data pattern is transmitted [116]. Polarization controller is widely used to solve the polarization mismatch problem between the SMF and the photonic integrated circuit (PIC). In Ref. [134], the polarization control scheme is adopted in the Microring-based WDM system to achieve the WDM polarization-independent receiver. In Ref. [135], the polarization controller is used in the coherent receiver to stabilize the polarization state of the local oscillator (LO). The polarization controller can be further improved by modifying the optical device performance and updating the controller algorithm.

*2. Nonlinearity compensation for PAM transmission.* Compared with NRZ, PAM4 can effectively increase the data capacity of a single channel, but it needs to effectively compensate for the deterioration of the eye diagram caused by static nonlinearity and dynamic nonlinearity. In Ref. [113], pre-distortion is used to adjust the electrical signal level to compensate for the distortion of the optical eye pattern caused by static nonlinearity, thereby generating a uniform optical eye pattern. Furthermore, feed-forward equalization (FFE) is used to compensate for level-dependent inter-symbol interference (ISI) caused by dynamic nonlinearity. In Refs. [136, 137], an optical digital-to-analog converter (ODAC) is adopted to generate a uniform eye diagram. This approach reduces the difficulty of driver design but makes the MRM design more complex. In these works, there are still many parameters that need to be manually selected, thus closed-loop automatic control methods are required.

*3. Compact modeling for photonic devices.* Since mature EDA tools have been able to support accurate simulation design for electronic circuits, most of the current work tends to model photonic devices on the EDA platform to combine mature electrical SPICE models to achieve accurate and fast photonic-electronic co-simulation. In Ref. [138], hierarchical design is used to model basic photonic devices, such as waveguide and coupler so that the MRR model can be combined through the basic photonic devices. In Ref. [139], the small-signal and large-signal model modeling of the MRM is realized through the SPICE model based on the coupled-mode theory. In Ref. [140], the S-parameter is used to describe the characteristics of the photonic device and the baseband-equivalent model is used to improve the simulation efficiency. However, the S-parameter based method is more suitable for modeling passive photonic devices. When modeling for active photonic devices, more metrics are needed to describe the characteristics of photonic devices, which undoubtedly increases the difficulty of modeling and reduces simulation efficiency. The baseband model can only reduce the frequency gap between the light and the modulation. Therefore, it is necessary to reduce the frequency gap between the modulation and the thermal fluctuation to further improve simulation efficiency.

### Concluding remark

Adopting new technological tools enables MRR-based transceiver arrays to meet the demands of data centers with high bandwidth and low power consumption. Therefore, the MRR-based transceiver array for co-packaged optics (CPO) is a promising solution to replacing the existing implementation of pluggable optical modules and become mainstream in the future [141].

**Acknowledgements**

This work was partially supported by the Open Project Program of Wuhan National Laboratory for Optoelectronics (No. 2021WNLOKF013).

## Mach–Zehnder modulator-based transmitter for co-packaged optics^7^

### Status

Mach–Zehnder modulator (MZM)-based transmitter has already been demonstrated in hybrid integration [142, 143] and monolithic integration platforms [144, 145]. In hybrid integration, the connection between the photonic chip and the electronic chip results in parasitics that limit the bandwidth and power consumption. 3D integration of electronic and photonic wafers performs with very low inter-connection parasitics capacitance. Monolithic integration further reduces the parasitics of the wiring, thus improving performance but also increasing manufacturing costs. For the modulator only, the EO bandwidth of a single MZM has reached 60 GHz [146] and the data rate of a single MZM has reached 240 Gb/s [147].

The MZM-based transmitter has made rapid progress in recent years. In 2006, Luxtera demonstrated the first fully integrated optical transmitter system in silicon technology platform [148] using current-mode logic (CML). Systematic design and simulation methodology was reported in Ref. [149]. A multi-segment transmitter that can potentially remove the power-consuming electrical DAC from optical links for low-cost applications was reported in Ref. [150]. To solve the large area consumption of passive inductors, Kim et al. realized an area-efficient modulator driver by adopting custom-designed shared inductors [151]. In 2016, IBM [152] demonstrated the first fully monolithically integrated silicon photonic four-level PAM (PAM-4) transmitter operating at 56 Gb/s with error-free transmission (bit error rate < 10^–12^) up to 50 Gb/s without forward-error correction. IHP reported a monolithically integrated Si MZM transmitter with the highest ER values (11 dB) at that time, which demonstrated the potential of the monolithically integrated transmitters based on the SE-MZM concept [145].

For hybrid integration, the single MZM-based transmitter has reached a data rate up to 100 Gb/s with 2.03-pJ/bit energy efficiency [143]. For monolithic integration, the single MZM-based transmitter has a data rate of 56 Gb/s with 4.8-pJ/bit energy efficiency [152]. In all these works, various techniques are adopted to maximize the system performance (data rate, ER, bit error rate, and energy efficiency).

### Current and future challenges

Several technological challenges remain to be tackled to further improve the performance and robustness of the MZM-based transmitter.

We outline these challenges below:

*1. High voltage swing driver.* Due to the limited breakdown voltage of the transistor, the voltage output of the electronic driver is generally insufficient for the MZM. In general, a SiP MZM has a *V*_π_*L product of approximately 1.5 V⋅cm [153, 154], suggesting that relatively long devices or large voltage swing are required to achieve modulation depth. For the same device length, if the voltage swing is not large enough, the difference between signal “1” and signal “0” will be small, resulting in low SNR and sensitive performance. Low voltage swing can lead to bit error since the transmitted signal is so weak that the receiver cannot distinguish it. Therefore, it is necessary to improve the voltage output swing of the driver. In the case of SiGe HBT technology, the breakdown voltage (BV) is typically ~ 2 V while maintaining a sufficient high ft/fmax, e.g., 300 GHz [155]. However, as for the advanced CMOS node, the BV of the core transistor is below 1 V [156], which means additional techniques should be used to improve the output voltage swing.

*2. High bandwidth.* Although a higher data rate requires higher bandwidth, the ft/fmax of transistors improves slowly. Thus, all kinds of bandwidth extension techniques are developed, such as inductive peaking [157–161], negative resistor, and capacitor splitting [162]. Also, equalization techniques, such as feedforward equalization (FFE) [65, 142, 163, 164] and continuous time linear equalization (CTLE) [165–167] are widely used in transmitters. Furthermore, distributed amplifier natively has wide bandwidth compared to lumped amplifier. Many works based on distributed amplifier structure have been reported, such as [65, 155, 163, 168–174], and [142]. The lumped driver is easy to implement but has limited bandwidth and modulation format. The distributed driver has superior operational BW, low sensitivity to components mismatch and modeling inaccuracies, as well as broadband power matching. However, it needs complex design, precise phase, and delay control.

*3. High energy efficiency.* Technology requirements for intra-DC optical interconnects are quite different from traditional long-distance telecommunication transport systems. Intra-DC interconnects have a much shorter reach (typically < 2 km) with a large number of connections, thus their cost is largely dominated by the transmitters. Additionally, intra-DC optics have more stringent power consumption, density, and cost requirements due to their sheer volume. Typically, voltage mode logic (VML) [142] is more power-efficient than current-mode logic (CML) [175–178]. Furthermore, single termination [149, 179, 180] can save half of the power consumption under the precondition of the same output voltage swing compared to the dual termination structure. However, the single termination scheme will introduce more reflection due to the impedance discontinuity. Another point to improve the energy efficiency is to employ a push–pull structure [65, 178, 181–183] rather than the pull-down structure with resistor load.

*4. Circuit-level photonic-electronic co-simulation.* MZM-based transmitter is a photonic-electronic integrated circuit [108]. Nowadays, photonic device design is mainly a device-level simulation implemented by PDA tools [129], while electronic circuits are circuit-level or system-level simulations implemented by mature EDA tools. Device-level simulation can provide very accurate simulation results but cannot be directly applied to circuit-level or system-level simulation design due to its low simulation efficiency. How to perform circuit-level or system-level photonic-electronic co-simulation is a prerequisite for MZM-based transmitter design.

### Advances in science and technology to meet challenges

Recent advances in photonic integration have addressed some of the challenges discussed above.

*1. Stacked CMOS output driver structure.* For SiGe HBT designs, BV doubler is widely used to overcome the voltage limit from a single transistor [178, 184–186]. The BJT is a current controlled device, while the MOSFET is a voltage-controlled device. The BV-Doubler for BJT can no longer work properly for MOSFET. Thus, stacked FET technique [156, 175, 187–192] replaces the empty of BV-Doubler. The stacked FET structure is commonly used in power amplifier designs.

*2. Inductive peaking and cherry-hopper pre-driver.* The distributed amplifier can provide large BW, but consumes large area and high power, and are difficult to design. Passive filtering (e.g., shunt and series peaking) has been used since the 1930s to extend amplifier bandwidth. It uses inductors to trade off between bandwidth and peaking in the magnitude response [193, 194]. Cherry-hopper amplifier is first used in 1960s [195]. It is widely used in optical interconnects. In Ref. [196], the cherry-hopper structure is used to enhance the bandwidth of the modulator driver. In Ref. [197], a 1-pJ/bit 80-Gb/s 2^15^ − 1 PRBS generator with a modified Cherry–Hooper output driver is presented.

*3. Compact modeling for photonic device.* Since mature EDA tools have been able to support accurate simulation design for electronic circuits, most of the current work tends to model photonic devices on the EDA platform to combine mature electrical SPICE models to achieve accurate and fast photonic-electronic co-simulation. In Ref. [138], hierarchical design is used to model basic photonic devices, such as waveguide and coupler, so that the MZM model can be combined through the basic photonic devices. In Ref. [198], the small-signal and large-signal model of the MZM is realized through the SPICE model. In Ref. [140], the S-parameter is used to describe the characteristics of the photonic device and the baseband-equivalent model is used to improve the simulation efficiency. However, the S-parameter based method is more suitable for modeling passive photonic devices. When modeling for active photonic devices, more metrics are needed to describe the characteristics of photonic devices, which undoubtedly increases the difficulty of modeling and reduces simulation efficiency. The baseband model can only reduce the frequency gap between the light and the modulation. Therefore, it is necessary to reduce the frequency gap between the modulation and the thermal fluctuation to further improve the simulation efficiency.

*4. Segmented driver.* The traveling-wave (TW) MZM requires a long phase shifter (2 − 3 mm) to obtain a sufficient optical extinction ratio due to the low modulation efficiency [199]. Thus, the electrode will introduce significant T-line attenuation. To satisfy the requirement of the ER, a higher voltage swing of the driver is demanded, e.g., 3 − 4 *V*_ppd_. Such a high voltage output swing is hard to implement using CMOS technology. Therefore, the lump-segmented (LS) MZM is developed to improve the modulation efficiency. By dividing a long phase shifter into multiple short segments and mapping the driver slice into each segment, the signal attenuation of each segment has been greatly reduced. Furthermore, each segment can be treated as a small capacitive load to the driver, resulting in higher power efficiency. Ref. [200] employed a simple and comprehensive modeling approach based on the microwave transmission matrix theory to analyze the frequency response of segmented traveling-wave optical EAM modulators.

Though LS MZM has many advantages, the choice of segment length, segment number, and the trade-off between the total power consumption and segment number will significantly influence the final performance [201].

Besides, timing matching becomes another important problem in segment configuration. Specifically, the electrical signal in different driver units should keep pace with the optical signal in different segment MZM units. Refs. [144] and [155] used artificial-designed T-lines to maintain the timing matching. Ref. [142] adopted the PI-based timing calibration approach, which can provide a wide-range delay adjustment of ~ 11 ps. The clock buffer can also provide a fixed delay (~ 10 ps) and the calculated optical propagation delay from adjacent segments is approximately 21 ps, which could be fully covered. Furthermore, considering the PVT varies, a calibration procedure of the optical eye diagram is also developed to further optimize the E-O velocity match.

### Concluding remark

Although there are still many difficulties, new techniques and innovations will constantly push the limit. Therefore, MZM-based transmitter for co-packaged optics (CPO) is a promising solution to replacing the existing implementation of pluggable optical modules and become mainstream in the future [141].

## Optical receiver front-end electronics in the CPO era^8^

### Status

As the aggregate bandwidth of the data center core switch reaches 51.2 Tb/s, pluggable optics has become increasingly incompetent [36] due to the limited space in the switch front panel and excessive channel loss between SerDes and switch IC. Therefore, co-packaged optics (CPO) has been proposed to unleash the future bandwidth bottleneck at data center. Using integrated optics and electronics, the pluggable optical module can be dissolved and co-packaged with the switch IC. Not only can the bandwidth density limit at the switch front panel be removed, the in-package millimeter-long electrical link can incur a much smaller loss, which will greatly ease the SerDes design and facilitate lower power.

**Fig. 15:**
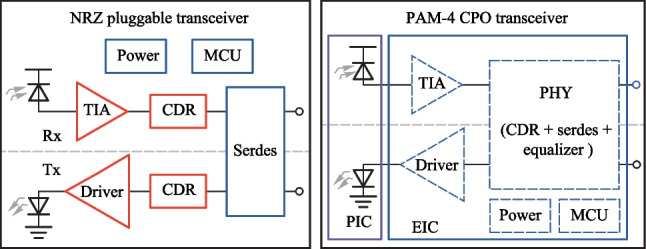
NRZ pluggable vs. PAM-4 CPO transceiver, red: SiGe, blue: CMOS, purple: silicon photonics

Meanwhile, as channel speed reaches 50 Gb/s and beyond, 4-level pulse amplitude modulation (PAM-4) has been introduced over non-return-to-zero (NRZ) to save half the bandwidth and the electronics in the optical module have been re-shuffled into its new form, as shown in Fig. 15. Most backend functions like CDR and SerDes are merged into a CMOS PHY chip (also known as DSP on some occasion), where intensive digital equalization is also brought in to compensate for the worsened signal integrity in PAM-4 signalling. Meanwhile, most high-speed front-end analog electronics (TIA/Driver) remain SiGe-based to leverage its superior analog performance. However, a monolithic approach is inevitable for CPO application that requires an extreme level of integration and power consumption, which will require the analog TIA and Driver to be integrated into the CMOS EIC chip. Here, we discuss the design issue of the TIA.

### Current and future challenges

Since CMOS is optimized for digital function, it exhibits natural advantages in power consumption and cost structure (at volume). However, it also shows weakness in analog performance. For CMOS based high-speed PAM-4 TIA to be integrated in CPO EIC, there are several critical design challenges.

*1. Noise.* Although the bandwidth is halved in PAM-4 signalling, the SNR also shrinks by three folds and the role of noise is further amplified. The noise floor of a TIA is inversely proportional to a circuit parameter known as the transimpedance limit [37], which is generally dictated by the technology speed expressed in the form of transit frequency (*f*_t_). Therefore, in SiGe-based design, faster technology with higher *f*_t_ is always preferred in implementing low-noise TIA. However, for CMOS TIA, more advanced technologies do not necessarily offer a dramatic boost in *f*_t_ when FinFET comes into play from 16 nm node and beyond [38]. To make things worse, it is often difficult to bias the transistor in its peak *f*_t_ condition due to several design constraints in CMOS [39]. Therefore, noise performance in CMOS TIA suffers.

*2. Bandwidth.* In general, high-speed optical communication circuits need the largest bandwidth over other applications, which keep pushing the limits of technology that foundries can offer. In this context, at least three factors make CMOS inferior compared to SiGe. First, the limited supply voltage (< 1 V) makes it difficult to build an effective buffer in advanced CMOS compared to SiGe, where the high-performance buffer (emitter follower) can decouple the gain stages and protect bandwidth [40]. Second, the low supply voltage also limits the maximum gain of each stage, as the transconductor transistor is biased at high current density for speed. Therefore, more gain stages are needed, inducing a severe bandwidth drop effect [41]. Finally, CMOS transistors have relatively small intrinsic device parasitics and large device footprints, making them very sensitive to extrinsic wiring parasitics, which can even dominate the overall parasitics. Together, these aspects make it difficult to design high-bandwidth circuits in CMOS.

*3. Linearity.* As data format evolves from NRZ to PAM-4, a new design consideration, linearity, comes into play. Non-linearity in PAM-4 signal will cause gain compression and an increased error rate. The difficulty resides in the fact that the input signal strength at the receiver side can vary more than 30 dB (1000X), rendering it difficult for the receiver to remain linear. On the contrary, at the transmitter side, the signal fed into the output driver stage is set by design, which alleviates the difficulty. Another issue comes from the direct contradiction between the reduced CMOS supply voltage (< 1 V) and the large receiver output swing needed to drive the following ADC stage, which can be several hundreds of millivolts strong, making the linearity difficult to maintain. An additional issue that also heavily impacts the PAM-4 signal integrity is phase linearity, to be clarified from the magnitude linearity discussed so far. Phase linearity is closely tied to the time-domain jitter, which can have a major impact on the decision circuit when the signal is transformed from analog to digital domain. Since there are 16 possible signal change patterns in PAM-4, the resulted jitter from phase non-linearity has a much severe adverse effect than that in NRZ.

### Advances in science and technology to meet challenges

*1. Noise reduction.* Since the transimpedance limit tends to be the key constraint that limits TIA noise, it is possible to reduce noise by tackling it. The first is a divide-and-conquer approach to circumvent the transimpedance limit, which decouples the bandwidth and noise goals and solves them sequentially [42]. The second type of approach manages to exceed the transimpedance limit by using cascaded multi-stage amplifier [39]. Of course, one can always combine the two approaches to reach an even better outcome. Moreover, the much lower capacitance from germanium photodiode (PD) in most silicon photonics platforms [43] can also greatly reduce noise [40], where a synergized co-design approach is needed [44].

*2. Bandwidth extension.* The most popular approach for bandwidth extension in CMOS is to use inductor peaking. Many sophisticated inductive peaking approaches have been developed at various stages of the amplification chain. At the pre-amplifier stage, bondwire inductance is often exploited to create series peaking with the capacitance from PD [40] to extend bandwidth. With on-chip inductors, many recent CMOS TIAs have reached a data rate of 53 to 64 Baud [45, 46]. Notice that with many inductive peaking, the transfer function tends to have in-band gain ripple or deteriorated phase linearity. Thus, the bandwidth extension at PAM-4 and linear application bears more care and simulation.

*3. Linearization.* Most linearization techniques rely on gain control at the topological level or source/emitter degeneration at the transistor level to reduce non-linearity. In the CMOS PAM-4 receiver, by tuning the gain of VGA (Variable Gain Amplifier) that follows the TIA [45], 2% of THD can be achieved up to a few hundred microamperes, which is on par or even better than those built with SiGe [47]. In the CMOS coherent receiver, the linearity is generally better due to the differential input characteristic. In Ref. [48], with the help of gain control and source degeneration in the pre-amplifier stage, less than 2% of THD is achieved with 1.8 mA_pp_ input current.

### Concluding remarks

As CPO will proliferate in the future 100 Tb/s era due to the imminent bandwidth bottleneck at data center, integration of front-end receivers with back-end processing electronics will become inevitable. This requires the technology for receiver design to shift from analog optimized SiGe to digital optimized CMOS, which will bring tremendous challenges. Possible design techniques to overcome problems regarding noise, bandwidth and linearity have been introduced here. As these techniques are combined with the instinct advantage of low power and low cost, monolithic CMOS electronics will fully unleash its power in the era of CPO.

**Acknowledgements**

This work was supported partly by the National Natural Science Foundation of China (Grant No. 62074126), National Key Research and Development Plan (No. 2020YFB2205801), Shaanxi Key Research and Development Plan (No. 2020GY-019), and Fundamental Research Funds for the Central Universities (No. XZY012020018).

## 2.5D and 3D advanced packaging for co-packaged optics (CPO)^9^

### Status

As data center traffic grows at an unprecedented rate driven by advances in Artificial Intelligence (AI) and Machine Learning (ML), network infrastructure must expand capacity while maintaining its total power consumption and footprint. Ethernet switches and optical devices also demand more bandwidth each year, with higher bandwidth density and energy efficiency [1–3].

**Fig. 16:**
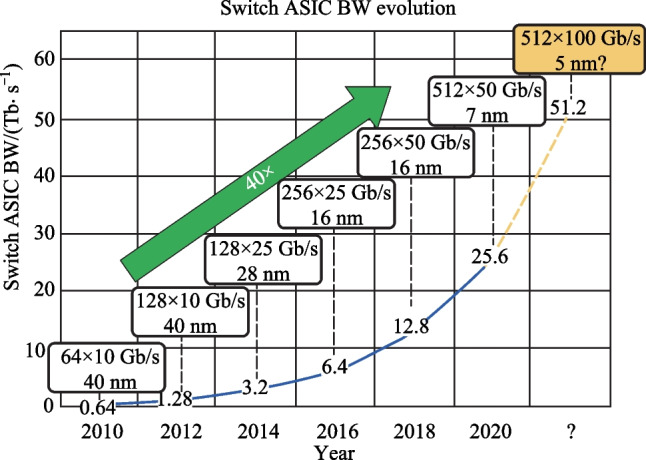
Bandwidth growth of Switch and ASIC in the last decade [4]

From 2010 to 2020, the bandwidth capacity of commercial switch integrated circuit (IC) increased by 40 times, from 0.64 to 25.6 Tb/s, while the process node of switch IC decreased from 40 to 7 nm, as depicted in Fig. 16.

If the transmission bandwidth of the switch IC per rack unit (RU) reaches above 25.6 Tbps, a RU needs 32 or more 800 Gbps face-plate-pluggable optical modules. However, the 800 Gbps pluggable optical modules with the current same form factor are challenging in terms of the required densities of electrical/optical connector, power consumption, and so on.

**Fig. 17:**
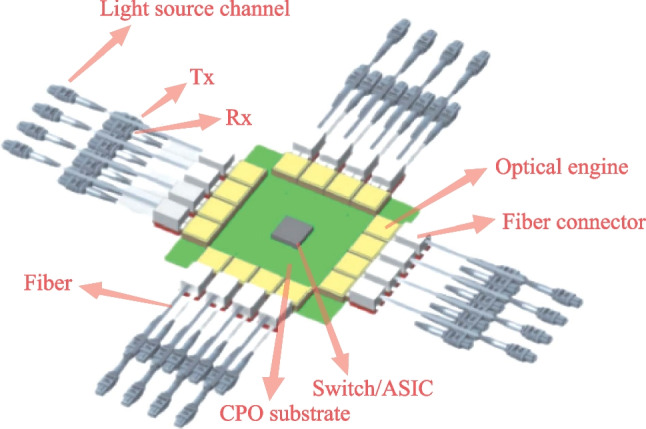
Full assembly of co-packaged switch, showing sixteen transceiver modules

Therefore, co-packaged optics (CPO) is regarded by many companies and experts as a key technology to achieve higher speed, wider bandwidth, and greater throughput [5, 6]. It can minimize the power of the electrical links to or from the optics and substantially increase the total escape bandwidth of the chip packages by offering an extra dimension for wiring additional chip pins. In March of 2021, OIF launched the 3.2 T co-packaged module project and developed a 3.2 T co-packaged optical module draft, defining Ethernet oriented switching applications. The mechanical layout of the CPO assembly is shown in Fig. 17 [7].

CPO solutions have been emerging in recent years. A collaboration between IBM and II-VI corporated to develop a chip-scale co-packaged optics module that can be directly attached to the top of an organic first-level package. They achieved energy efficiency of < 4 pJ/bit (16 channels) and the data rate is 56 Gb/s NRZ [8].

Ranovus and IBM launched 51.2 Tb/s optical modules based on CPO technology. They developed fiber V-groove interconnect packaging technology that utilizes passive alignment capabilities to achieve low insertion losses across a wide spectrum of O and C bands [9].

Intel has designed, developed, and demonstrated a SiPh (Silicon Photonics) IC-based transmitter and receiver suitable for optical co-packaging with switch ASICs. It has a compact form factor capable of bandwidth density > 40 times that of 100 Gbps QSFP28 pluggable optics, and has a power efficiency of < 20 pJ/bit and shows a path to 13.5 pJ/bit [10].

Ayar-labs and Intel made the first CPU integrated with optical I/O in package. They used O-Chiplet and EMIB technology to achieve < 5 pJ/bit energy efficiency and more than 1Tbps/mm bandwidth density [1, 11].

**Fig. 18:**
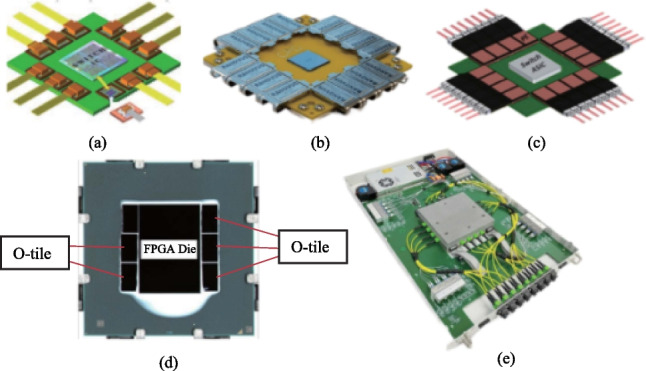
CPO solutions. **a** IBM and II-VI. **b** Ranovus. **c** Intel. **d** Ayar-labs. **e** Hengtong Rockley

Hengtong Rockley has developed a 3.2 T CPO working prototype based on silicon optical technology, which shortens the distance between photoelectric conversion function and core switch chip [12]. These productions are shown in Fig. 18.

### Current and future challenges

The CPO is capable of high-capacity transmission, but still faces a series of challenges.

*1. Light source.* The integration of laser source has always been the difficulty of photonic integrated circuits. As we need to make light propagate inside CPO, the light from the light source passes through the fiber and enters the optical chip and other components through a specific coupling mode. It is necessary to ensure that the transmission of light is stable, convenient, and reliable. Whether to use on-chip laser or external laser is determined by a variety of factors, such as operating temperature, laser reliability, and SiPh process platform compatibility [13].

*2. Coupling structure.* The coupling boils down to a huge size mismatch between the fiber core and the Si waveguide, causing considerable optical transmission loss when light emitting from the fiber core enters the waveguide directly [14]. In most cases, we can achieve low coupling loss (CL) by edge coupling, grating coupling, and evanescent coupling. Each method inevitably requires designing a suitable spot size convert (SSC) to match the waveguide.

*3. Thermal management.* The power consumption of the switch die is much higher than that of the CPO module. Thermal crosstalk on the CPO module from the switch die is a severe challenge. Meanwhile, some optical devices in PIC (Photonics IC) are sensitive to temperature, especially the switch die with large power consumption, or on-chip laser integrated in close proximity to PIC. Baehr-Jones’s group [15] fabricated micro-ring structure with a radius of 30 μm on SOI substrates and tested their temperature sensitivity. When the temperature was increased from 20 to 28 °C, a shift of 0.4 nm was found in the transmission spectrum, while the insertion loss changed by up to 9 dB.

*4. High-speed interconnect.* Compared with traditional packaging solutions, CPO requires higher data transmission speed, but the existing packaging structure cannot achieve high-speed and high-density interconnection. 2.5D Si interposer and 3D chip stacking are the main technology to achieve the target [16].

### Advances in science and technology to meet challenges

Recent advanced solutions based on these challenges have been proposed.

*1. Inside or offside laser integration*. We have summarized three methods that roughly include off chip laser, attached laser, and bonded hybrid laser. Although the off chip laser is flexible and substantially reduces the power of the CPO, it may require accurate alignment for large area optical interconnection and expensive long-term polarization-maintaining fiber (PMF) [17]. The most representative technology for the attached laser is that the laser is mounted to the PIC by flip-chip, and the light is guided into PIC by the edge coupler. As for bonded hybrid laser, there are three solutions, specifically directly bonding, insulation bonding, and metallic bonding [18].

*2. Multi-tip and overlapped taper’s structure.* Now several research groups have developed the optical coupler that achieved less than 1 dB CL. For example, Takei’s group launched a knife-edge shape coupler of 0.21 − 0.35 dB CL [19]. Papes’ group designed a large mode size for Si photonic wire waveguide coupler of 0.75 dB CL [20]. Picard’s group proposed a structure of Si taper that is under multiple SiN rods and cladded by the SiON, and it achieved 0.5 − 0.9 dB CL by verification [21]. In addition, the photonic wire bonding (PWB) technology no longer needs the traditional high-accuracy alignment between the large-sized optical fibers and the waveguide. Christian Koos and his team connected Indium phosphide (InP)-based horizontal cavity surface emitting lasers (HCSELs) to passive silicon photonic chips by PWB, which achieved the lowest insertion loss of 0.4 dB at that time [22].

*3. Liquid-cooling and special structure cool plate.* In recent years, some companies have announced several methods for the thermal management of the optoelectronic integration. Intel’s liquid cooling solution is for 540 W of switch IC and 56 W of each CPO. According to the conditions given in the article, they lowered the temperature of the EIC (Electronics IC) by 35°C and the switch die by 8°C [1]. Cisco’s heat sink and cold plate assembly is a great plan [23]. Compared to forced-air cooling, liquid cooling has a more pronounced cooling effect, such as less noise, more uniform heat dissipation, and does not require an additional power supply to power the fan. In addition, cold plate with thermal interface materials and a heat spreader or heat sink can be combined with the liquid cooling solution for possibly better performance.

**Fig. 19:**
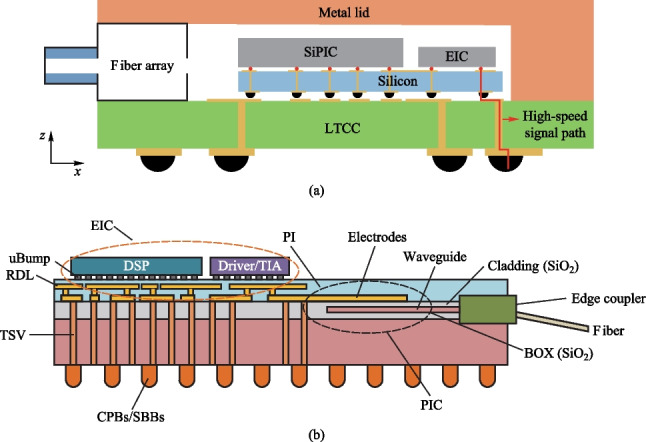
Optoelectronic 3D integration cross section **a** 2.5D solution. **b** 3D solution

*4. 2.5D&3D packaging of CPO module.* Institute of Microelectronics of the Chinese Academy of Sciences (IMECAS) has been dedicated to the research of advanced packaging technology. As for the packaging form of the CPO module, IMECAS has developed 2.5D&3D packaging technology. On one hand, the solution for 2.5D packaging is that silicon PIC and EIC are flipped on 2.5D silicon interposer, and secondary packaging is then used through a low-temperature ceramic substrate [245]. On the other hand, there are two options for implementing 3D packaging. First, the EIC is placed on PIC by micro-bump (pitch: 40 μm) and the stacking structure on PCB through wire bonding. Second, the PIC is integrated in the active photonic interposer while retaining the passive function. TSV (10 μm × 100 μm) and redistribution layer (RDL) are then fabricated on the basis of EIC and the EIC is attached to the interposer face to face. Finally, the substrate and interposer will be connected by a solder ball (pitch: 150 μm). Our high-frequency transmission line shows an excellent insertion loss at 67 GHz [24]. The 3D solutions are shown in Fig. 19.

### Concluding remark

In recent years, the research and development of CPO technology is more active due to the increasingly evident advantages of photonic chips in aspects like data transmission and high-speed interconnection. It is expected that CPO technology would replace the pluggable optical module. There are still some challenges, including the future application is still very extensive.

**Acknowledgements**

The authors wish to thank and acknowledge support from Key Research and Development Program of Jiangsu (No. BE2022051-3).

## Electronic-photonic co-simulation for co-packaged optics^10^

### Status

Electronic-photonic co-simulation is an important condition for the future large-scale commercialization of co-packaged optics, which can greatly improve design efficiency. There are many published results for electro-optical co-simulation from academia [108, 138, 202–204]. In Ref. [203], the basic optical device is modeled by Verilog-A and SPICE language, which can be simulated with electrical circuits. In Ref. [138], the primitive component of the optical device is modeled with Verilog-A language, so the composite device and optical-electrical interactions can be simulated. Many companies have also launched their simulation tools. Synopsys has a set of co-simulation software. The optical device can be simulated in Sentaurus or Rsoft, while the whole optical system can be simulated in OptSim or ModeSYS. The electronics and photonics can be simulated together in OptSim with PrimeSim HSPICE. The electro-optical co-simulation can be also achieved using softwares from different companies. The Ansys Lumerical can achieve optical device simulation with FDTD, MODE, and FEEM kits and the Interconnect kit can achieve simple electro-optical co-simulation by itself. It can handle complex electro-optical co-simulation with Cadence platform.

At present, photonic design automatic (PDA) tools are mainly based on device-level simulation, which can provide accurate photonic device-level simulation. However, its simulation efficiency is very low, thus is not suitable for large-scale circuit-level or system-level simulation. At the same time, electronic design automatic (EDA) tools are mostly based on circuit-level or system-level simulation. Therefore, the photonic device models that can be applied to circuit-level and system-level simulations are the key to large-scale electronic-photonic co-simulation. Modeling photonic devices on EDA tools has proven to be an effective solution to enabling electronic-photonic co-simulation [108, 138, 202–205]. Verilog-A language can describe the physical properties of photonic devices well and has good compatibility with SPICE models, thus is widely used in photonic device modelling [138, 202–204, 206, 207]. The photonic device model based on Verilog-A has good scalability due to the hierarchical design procedure. However, we find there are still certain problems in practice with Verilog-A modeling, such as the simulation process being prone to non-convergence and the inability to describe complex signals [138]. The SPICE models can also be used to describe the physical properties of photonic devices [139, 205, 208, 209]. It is shown that the description of the physical properties of a specific photonic device can be achieved through a simple RLC network, resulting in high simulation efficiency. The existing SPICE photonic device models have strong specificity but poor scalability [205], so it is difficult to cope with the current complex photonic device links. Since the current PDA tool can derive the physical characteristic curve of the photonic device through S-parameters [140, 210], it can describe the photonic device very accurately. The S-parameter modeling method can be well applied to the modeling of passive photonic devices. Since we need to obtain the S-parameter matrix of the photonic device in different states, it is not suitable for active photonic device modeling. The Simulink tool based on MATLAB has been proven to model photonic devices well [211, 212], but it may not be suitable for large-scale electronic-photonic co-simulation due to the better compatibility with the EDA than Verilog-A and SPICE model.

### Current and future challenges

To further improve the accuracy and simulation efficiency of electronic-photonic co-simulation, there are still some technical challenges to be solved. We outline these challenges below:

*1. Compact optical model*. The accurate optical device model should contain both transient behavior and spectral behavior. The spectral behavior is easy to model since many simulation tools can simulate the wavelength-dependent properties. However, the transient properties are hard to acquire for several reasons: the high frequency of the optical carrier makes it hard to achieve long-term simulation and monitor the transient results; the transient performance is influenced by multiple dynamic factors that are difficult to simulate, such as the bandwidth of the thermo-optic phase shifter in silicon photonics; the interaction of adjacent device, such as thermal crosstalk. In previous works, the spectral properties are always modeled precisely and match well with the measurement results, but the transient properties are less considered.

The optical signal contains multiple dimension information, such as wavelength, magnitude, phase, polarization, and mode. In a real system, the information in different dimensions will change dynamically. The magnitude of the optical signal transmitted through fiber will decrease due to intrinsic loss. The polarization state of the wave will change due to the asymmetry of the fiber and external disturbance [213]. The mode will be strongly mixed arbitrarily during propagation [214]. These time-varying properties will strongly affect the performance of the optical system, making it essential to model these effects in simulation tools. Most of the previous work only focuses on the wavelength dimension, magnitude, and phase. Several works contain polarization information, but dynamic properties have not been considered [203].

The nonlinearity effect will always take place in case of high light intensity, which is the common case in an on-chip cavity, such as the micro-ring. The two-photon absorption (TPA) effect and the free-carrier absorption (FCA) in microring will change both the carrier density and temperature, which will introduce the bistability of the microring [215, 216]. That effect will strongly influence the property of the device and must be modeled precisely, which is less considered in recent works.

*2. Time-domain simulation.* Time-domain simulation plays an important role in electronic-photonic co-simulation. The most critical issue in time-domain simulation is how to improve simulation efficiency while ensuring simulation accuracy. Unfortunately, time-domain simulations are often inefficient in many cases. For example, in the application of wavelength locking of the micro-ring modulator, there is a large mismatch between the optical signal (~ THz), the electrical modulation signal (~ GHz), and the thermal fluctuation frequency (kHz). To ensure the simulation accuracy, we must determine the transient simulation step size (~ ps) according to the frequency of the optical signal and determine the total simulation time according to the thermal fluctuation frequency (~ ms). This results in a very long simulation time and low simulation efficiency. In addition, many photonic devices cannot work independently from the control circuit, so closed-loop time-domain simulation is the key to realizing the stable operation of photonic devices. As a result, the modeling scheme of photonic devices such as s-parameter is not well suited, and a compact continuous-time photonic model is required to realize closed-loop time-domain simulation.

*3. Frequency-domain simulation.* Frequency-domain simulation is also an important step in electronic-photonic co-simulation. To obtain the spectral characteristics of photonic devices, the photonic device model with frequency-domain simulation capability is needed. Although it is possible to obtain the output value at each frequency point through time-domain simulation, obtaining the frequency-domain curve with plotting points requires a long simulation time. Therefore, fast frequency-domain simulation is also the key to improving the efficiency of electronic-photonic co-simulation.

### Advances in science and technology to meet challenges

Recent advances in electronic-photonic co-simulation have addressed some of the challenges discussed above.

*1. Hierarchical photonic device modeling.* To improve the scalability and accuracy of photonic device models, the idea of hierarchical modeling is proposed to realize photonic device modeling. Complex photonic devices consist of basic photonic devices, such as waveguides, couplers. Therefore, the detailed description of the basic photonic device model by Verilog-A can ensure the accuracy of the photonic device model [138, 202]. Furthermore, by connecting basic photonic devices, complex photonic devices, such as MRR and MZI, can be combined, and even more complex photonic links and systems can be generated. Although hierarchical modeling ideas are highly scalable, simulation efficiency is a key limit compared to a dedicated photonic device model [205]. Therefore, how to further improve the simulation efficiency of hierarchical photonic device modeling is the key in the future.

*2. Equivalent baseband model.* Frequency mismatch between optical signals, electrical modulation signals, and thermal fluctuations results in lower simulation efficiency. Based on the principle of the baseband signal, we can carry the optical signal to the baseband [138, 210], thereby transforming the signal of the THz-level into GHz-level, shortening the frequency mismatch between the optical signal and electrical modulation signal. The simulation step size can be adjusted to the 10 ps-level, thereby reducing the simulation time. Therefore, the equivalent baseband model can improve the simulation efficiency by shortening the frequency mismatch. However, the frequency mismatch between the electrical modulation frequency and thermal frequency is still large and will be a key factor limiting the simulation efficiency in the next step.

*3. Chirp frequency simulation.* It is very important to obtain the frequency-domain response of the optical device efficiently. Since most of the existing photonic device models are time-domain models [108, 138, 205], the frequency response of photonic devices cannot be directly obtained. The traditional method obtains the frequency response of the time-domain photonic device model at different input optical frequencies. Finally, the frequency response of the photonic device is obtained by combining these frequency points. This method requires time-domain simulations at multiple frequencies, which is undoubtedly time-consuming. To obtain the frequency response of photonic devices more efficiently, a broadband analytic chirp excitation method [204] is proposed to estimate the frequency response in a single transient simulation. The chirp signal has a fixed field magnitude and a time-varying phase term that depends on the instantaneous frequency. The frequency response of the system can be calculated by the spectrum of the output of the system and the spectrum of the input signal from one transient simulation. This method will greatly reduce the simulation time of the frequency response.

### Concluding remark

Employing the new techniques described earlier allows electronic-photonic co-simulation to meet the requirements of large-scale electronic-photonic co-design for co-packaged optics. However, there are still many unresolved problems. It is necessary to further optimize the simulation efficiency and simulation accuracy to meet the more demanding simulation requirements in the future.

**Acknowledgements**

This work was partially supported by the Open Project Program of Wuhan National Laboratory for Optoelectronics (No. 2021WNLOKF013).

## Considerations on photonic interconnect for HPC: a system point of view^11^

### Status

Photonic interconnect has become an important part of the networking solution in high-performance computing datacenters (HPC DCs), where there is a never-ending thirst for high bandwidth and pursuit of low latency. Optical transceivers have accompanied DC in the evolution of data IO from the era of 10 Gb/s to beyond 400 Gb/s and the link reach growing from 50 m to 10 km + . In the past five years, optical IO technologies have evolved towards a higher level of integration to catch up with the bandwidth growth of switch ASICs, and are being positioned closer to reduce latency and high loss of electrical signal at a high data rate (100 Gb/s). Such optical IOs, known as co-packaged optics/Near-packaged optics (CPO/NPO), have attracted investment from the datacom industry, hoping to achieve higher networking bandwidth at lower power consumption, latency, and cost. Multiple industrial prototypes have been proposed and showcased by key players, such as Intel [11], Hengtong Rockley [49], and Broadcom [50].

The IO bandwidth of each port in a photonic link is scalable using multiple wavelength-division-multiplexing (WDM) channels, offering an effective method of transmitting large data flow. Unlike electrical switches, optical switches build definitive optical paths directly between endpoints, thus offering lower latency and less power consumption by eliminating the use of multiple optical transceivers and the possibility of congestion than in electrical switches. These benefits of optical switching place high expectations on photonic interconnect for it to take on more roles than merely being the endpoints of the physical link in HPC networking solution. Copious research investigating the possibility of deploying optical circuit switching (OCS) in HPC were supported or led by HPC service providers. Both Facebook [51] and IBM [52] looked into networking architectures using hybrid electrical and optical switches to combine the flexibility of electrical switching and the large port bandwidth of optical switching, hoping to reduce cost and power consumption. Microsoft initiated the Sirius project to investigate the role of nanosecond optical switching technology in confronting the challenge of explosively growing network traffic and stringent latency requirement in HPC DC. In Ref. [53], a system-level demonstration was reported for a four-node cluster with performance approximating that of an electrical switch system but at over 70% reduced power consumption. The nanosecond switching of optical paths was carried out by the fast changing of the carrier wavelength by switching on and off an array of DFBs. In the following work reported in Ref. [54] in 2021, an optical frequency comb was employed to build an OCS network interconnecting 64 TORs via an AWGR. Both wavelength switching methods become expensive in terms of cost and power consumption as the required number of wavelength channel scales up with the node count. For a practical wavelength switching network to work out, a fast tunable laser, capable of ns-switching speed, wide wavelength range, and simple architecture, is still in urgent need. Nevertheless, these pioneering works led by industry players have showed confidence in photonic interconnect being a significant part of future HPC networking solution for high bandwidth, improved power efficiency, and low latency.

### Current and future challenges

*1. Next generation Ethernet rate: 200 Gb/s per lane*. HPCs are always leading in the deployment of new advanced optical transceiver technologies to achieve higher IO bandwidth, such that the computing power of GPUs can be more efficiently exploited. With optical transceivers based on 100 Gb-PAM4 being commercially deployed for a full range of use cases in DC, the focus of the next generation optical IO for short-range interconnect is now on 200G-based IM-DD technology. Assuming a PAM4 modulation format, 200 Gb/s requires 50 GHz + component bandwidth and increased dispersion penalty allowance from the optical link budget, pushing the limit of both components and equalizer algorithms. To date, the few demonstrations of 200 Gb/s optics are laboratory based [55–57]. It is foreseeable that more challenges await down the road, such as managing the power consumption to fit in the thermal management capability of form factors, improving the transmitter and receiver performance to make room in the link budget for reliability and longer reach, incorporating the 200 Gb/s in CPO/NPOs, etc. These challenges call for the development of high-bandwidth modulator, advanced electro-optical packaging technology sustaining the bandwidth of individual components, energy-efficient driver technology, integrated photonic components for dispersion compensation, advanced DSPs, etc.

*2. Speeding up, scaling up, and managing an OCS network*. The benefits of low latency, high IO bandwidth, and low power consumption brought by the OCS network have intrigued researchers to investigate the possibility of building the next generation HPC network using optical switching technologies. However, the large O (1000) nodes interconnect demand of HPC raises an immense challenge to maintaining the advantages of OCS network.

On one hand, simultaneously speeding up and scaling up of the OCS network is yet to be proposed at an affordable cost. A single MEMS-based spatial switch could support more than 300 × 300 port capacity but only offer switching time in the millisecond scale. This sub-second switching speed limits it to serve for low-frequency topology engineering as a supplementary switch path in addition to the electrical switches as did in Helios [58] and HOSA [52]. Photonic switches have been demonstrated to have us- or ns-switching speed, depending on the working physics employed [59, 60]. However, it exhibits rapidly growing insertion loss with the switch radix and non-uniform path loss associated with the topology and route. The advantages of fast switching and μm-scale size of the photonic switch will be jeopardized by a bulky, slow, and power-hungry control circuitry. In Ref. [61], the peripheral circuit was monolithically integrated with the photonic device for an 8 × 8 switch. However, scaling up to large radix is yet to be demonstrated. These characteristics put a challenge in scaling up the photonic switch to meet the radix demand. Using wavelength-dependent routers and light sources capable of nanosecond wavelength tuning, a fast and scalable OCS network may be within reach, but still awaits the industry to bring down the cost of the DWDM optical components.

On the other hand, unlike electrical switches, optical switching does not have a buffer to store data before redirecting. Therefore, it is unsuitable for IP protocol and does not support hashing techniques to handle congestion. Hence, the OCS network needs to be fully and universally managed to avoid conflict of traffic flows in both the space dimension and wavelength dimension. Network routing schemes and algorithms tailored for OCS network’s zero-buffer characteristics are being actively discussed in the community. Our group reported in Ref. [62] a wavelength assignment algorithm for a WSS-based all-optical rearrangeable Clos network to deliver Ring All-reduced service. The performance of such managing algorithms still needs to be further studied in larger clusters mimicking true world DCs. Another commonly overlooked problem is the physical implementation of the routing strategy, i.e., the controller of the optical switches. A central controller may seem feasible, but is difficult to implement with satisfying performance due to reasons, such as the massive connectivity of the controller to switch, prolonged time of route calculation, and non-uniform latency of control signal arriving at the distributed switching units.

*3. End-to-End switching time*. Although photonic switching technology has been reported ns-speed, the actual time required to cutoff an existing photonic interconnect link and build a new optical route consists much more than the mere switching time of photonic switches, including the decision making of the optical route, latency of photonic switches taking into account of its actuating electronics, time for the command to reach the controller from a central managing agent, and clock data recovery time. Since the decision making and the control latency are relatively fixed values, the action can be conducted prior to the switching overlapping with the previous data transmission period, so that this part of switching time can be theoretically reduced down to zero. However, the receiver locking time is inevitable and could easily consume up to a millisecond due to the varying and random phase and amplitude of the optical signal transmitting distinctive photonic links, unless burst-mode CDR and burst-mode TIA are employed. Researchers from IBM and UCSD have reported end-to-end switching time of < 382 ns in Ref. [63] using nanosecond SiP switch and burst-mode receivers. They have further shown in Ref. [64] that the system switching time can be further reduced to < 90 ns by incorporating a synchronizing control scheme of the photonic switch and burst-mode transceiver.

### Advances in science and technology to meet challenges

Facing the challenge of 200 Gb/s beyond, it is the right time to think holistically about the physical link and design the electrical and optical component synergistically, leveraging one’s perk to compensate for the other’s pitfalls, such that an economic solution can be acquired. Co-design of the electrical and photonic components has been exploited in research works on MZM and MRM to achieve higher IO bandwidth, lower power consumption, and smaller footprint [65, 66]. In Ref. [67], an integrated SiP transmitter capable of 100 Gbps On–Off-Keying operation with a power efficiency of 2.03 pJ/bit was reported by co-designing the SiP MZM and its CMOS driver. The driver provides a peaking effect to enhance the bandwidth of the MZM, allowing for more design space to mitigate the metrics of MZM instead of being forced to a trade-off among bandwidth, insertion loss, footprint, and modulation efficiency. This holistic design strategy has also been employed in Ref. [68] by researchers in Intel to enable 112 Gb/s PAM4 data transmission using an MRM. A driver was specifically designed with voltage-mode DACs to compensate for the MRM’s nonlinearity. The synergetic design strategy is also facilitated by foundry service providers, such as Global Foundries [69] and IHP [70], both offering monolithic electronic-photonic integrated circuit processes along with some co-design PDKs.

As stated in the previous section, CDR time has become a key contributor to the overall latency in a switching event using ns-switching optics. To address this issue, Microsoft has collaborated with academic researchers to investigate optical caching technologies to reduce the CDR time for OCS networks. In Ref. [71], a clock phase caching technique was employed in a synchronized network to reduce the CDR time down to sub-nanosecond. An optical frequency comb was employed for distributing the clock signal to align the clock frequency. The clock phase of all possible transmission links was measured and stored locally in the logics, and applied to the transmission data according to the optical link in use.

### Concluding remark

The non-stop pursuit of higher IO bandwidth, lower latency, and continuous cost reduction and power saving awaits for the maturation of full photonic interconnect networking solution for HPC. With the devotion to energy-efficient photonic engines, fast and scalable optical switching technology, and the network managing algorithm, the turning point is perhaps just a final push away.

## Optoelectronic hybrid interface in HPC interconnect^12^

### Status

High-performance computing (HPC) generally refers to large, fast, and efficient calculation. It solves challenging problems in the big science, such as cosmic science, nuclear science, life science, and meteorology, the large engineering, such as petroleum exploration and large-scale machinery manufacturing, the informatization, such as virtual city and unmanned manufacturing, and the entertainment industry, such as animation rendering and special effect synthesis. Recently, rapidly developing AI has become another important application scenario for HPC as its training process is hungry for computing power. For nearly half a century, the performance of HPC has been rapidly increasing at a rate of 1,000 times per decade, and the leading system has already reached the exascale (10^18^) goal. In the post-Moore’s Law era, the performance development of processor cores have reduced and the modern HPC systems adopt parallel computing architecture with heterogeneous processors. The component processors and memories connect, communicate, and collaboratively compute with each other through its interconnect.

The high-performance interconnect is the core component and important infrastructure of an HPC system, which is one of the most important factors affecting HPC performance and scale expansion. As the number of computing cores in a system grows, reaching tens of millions, the network scale needs further expansion. Based on the parallel computing mechanism, the computing core needs to collect calculation results of other cores for further processing. As a result, the communication cost has a great impact on the performance of a parallel computing system. To cut the data movement cost, network architects reduce the number of hops in the network by shorting the network diameter through topology innovation and switch-order enhancement. For transmission system designers, the primary task is to increase bandwidth and reduce latency. In addition to scale, bandwidth, latency, and network diameter, power consumption, reliability, and cost are also important figures of merit for interconnect networks.

With the link rate as the intergenerational milestone, the high-performance interconnect technology develops towards high order. All of the mainstream interconnects of high-performance computing systems on the Top500 list in November 2021 [76] evolved from the enhanced date rate (EDR) at 28 Gbps to the high date rate (HDR) at 56 Gbps. In recent years, Broadcom, one of the Ethernet leaders, iterates its Ethernet switch series, Tomahawk, one generation every two years. In 2020, it released its 400GbE switch with a throughput of 51.2 Tbps at a link date rate of 53.125 Gbps [77]. In terms of the most popular interconnect in high-end HPC InfiniBand, its main vendor Mellanox, acquired by Nvidia in 2019, even released in 2021 the Next Date Rate (NDR) InfiniBand switch, Quantum-2, at 112 Gbps [78]. The switch supports up to 64 400 Gbps ports and provides an average switching delay of only 90 ns.

### Current and future challenges

At the beginning of this century, when the transmission link date rate was only 5 Gbps (DDR, double data rate), optical technology had already been applied in communication networks in both metro long-haul telecommunication and warehouse systems. From then on, active optical cables (AOC) and optical transceivers have gradually completely replaced copper cables in intra/inter-rack interconnects and formed an optoelectronic hybrid interconnection network architecture based on electrical switches and optical interconnects. The main reason for copper displacement is that the electrical reach decreases as the link date rate increases due to the electrical transmission loss mechanism. However, optical transmission has no such problem because of its high carrier frequency.

Within such an architecture, data is processed and routed all by the electrical chip, while optical technology just offers an option of the electrical link with an additional photoelectric and inverse conversion. The additional conversions result in additional power consumption and latency. As a result, optical technology is always the last choice. All over the past 20 years, optical researchers have been trying to further replace electrical interconnect by placing optical engines on board near package [79], in package [80, 81], or even on chip [82, 83]. However, the quad small form factor pluggable (QSFP) edge-mounted AOC or transceiver with the medium reach (MR) electrical interface remains the mainstream of optical interconnects, even though the link data rate has already climbed up to 112 Gbps, and computing systems based on the on-board optical engine was demonstrated by IBM for over a decade [84]. The main reason is that the electrical interface technology is still advancing. Even in the NDR era, the long reach (LR) electrical interface can still cover 35 dB link insertion loss. In practice, shortening the electrical reach does not realize the potential of system power reduction. The biggest obstacle is the technical inertia of the switch chip architects. The switch chips used in HPC systems generally take advantage of the most advanced technology at the time with no design reference, and the cost of failure is staggering, no matter in capital or in labor. Therefore, chip architects disgust architecture with little compatibility. As a result, the LR interface is always the first candidate if it is acquirable, despite its higher power consumption. The situation is the same on the optical engine side. Its electrical interface follows the MR standard, as the chip inside is compatible with the edge-mounted scenario at a cost of higher power. Since power consumption on both sides remains the same, traditional optoelectronics integration does not reduce transmission power, though it has potential to do so.

However, the edge-mounted solution is unsustainable due to the limitation of front-panel bandwidth density. Though two types of package form, the QSFP-DD (double density) and the octal small form factor pluggable (OSFP) module, are developed to double the panel bandwidth density, the 1U standard rack panel can only support, at most, 32 such ports.

### Advances in science and technology to meet challenges

**Fig. 20:**
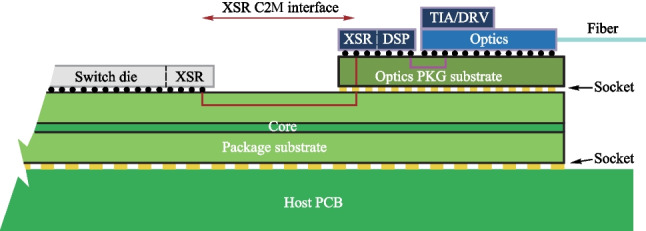
Schematic diagram of XSR-AUI CPO [85]

Although few large-scale application, three optoelectronic hybrid integration solutions have been developed, including near-package optics (NPO) [79], co-packaged optics (CPO) [80, 81], and monolithic integration [82, 83]. Among them, CPO is the most promising solution in the next generation 100G applications. The extra short reach (XSR) attachment unit interface (AUI) solution, as shown in Fig. 20, is the most popular proposal. The optical engine with an integrated digital signal processor (DSP) is co-packaged with its host as a whole through an XSR chip-to-module (C2M) interface. The solution, evolved from the NPO technology, is just a formal integration that compromises with the commercialized technology and hardly reduces the system power because of the reservation of the common electrical interface (CEI).

**Fig. 21:**
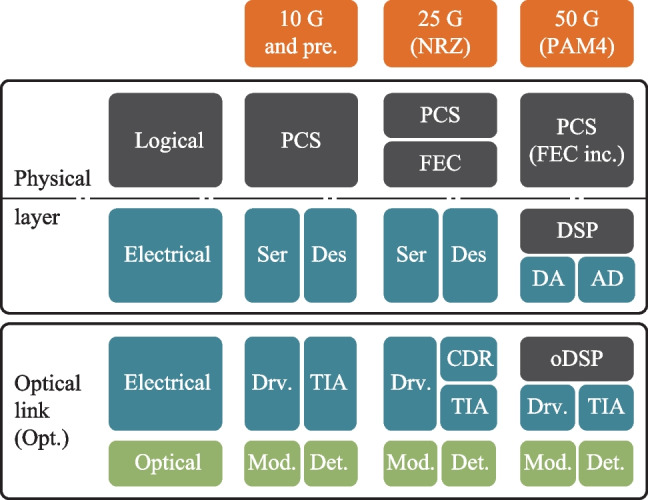
Development of physical layer

This CEI boundary separates the optical engine out of the collaborative optimization of very large scale integrated circuit (VLSI), though the optical engine is composed of some analog or even digital circuits. Some of these circuits, usually highly energy-consuming, are redundant on both sides of the CEI boundary. The existence of CEI has a historical inheritance reason that can be traced back to the very beginning of electrical interconnect, as shown in Fig. 21. However, in the 50G era, when 4-level pulse amplitude modulation (PAM4) becomes mainstream of signal modulation instead of non-return-to-zero (NRZ), the architecture is no longer suitable for the latest technology trends, such as the DSP + ADC (Analog to Digital Converter) architecture serial interface SerDes and the oDSP-based optical engine.

**Fig. 22:**
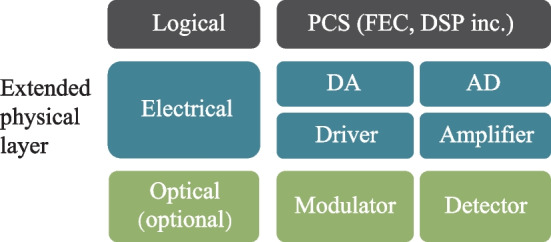
Architecture of the extended physical layer

In the era when optical link is no longer an option but a must in a system interconnect, a deep integration of optoelectronics requires a new interface framework to incorporate the optical engine into the VLSI system (Fig. 22). The extended interface physical layer architecture must achieve the two goals of simplicity and compatibility. On one hand, it must degenerate and compress the protocol flow of the overall optical interface. On the other hand, it must be fully compatible with independent electrical interface application scenarios.

To cover these two requirements, it is proper to integrate both the equalization algorithm for the electrical link in the SerDes DSP and that for the optical link in the optical engine oDSP, within the original physical coding sublayer (PCS). Thus, the new physical logic sublayer implements all functions related to the signal quality of the physical link, such as equalization and error correction. It can be configured to match the different signal quality to support different application scenarios. In terms of cost, the DSP function in SerDes is similar to the oDSP function in the light engine, and the cost of implementing full-featured integration is not high, only adding about 5% of the area. The new physical electrical sublayer contains all the remaining analog circuits, including the AD/DA in the SerDes, as well as the driver and amplifier in the optical engine. The new physical electrical sublayer can unify the upper-layer digital interfaces of various analog front-ends, which can realize the interchange of different electrical front-ends without affecting the upper-layer digital logic. A new physical optics sublayer is added for better optoelectronic hybrid co-design. It is optional to support a fully electrical interface.

### Concluding remark

In the foreseeable future, optical interconnect will become mainstream of high-bandwidth inter-chip communication. Not only is the evolution of existing technology unable to achieve a potential reduction in power consumption, but also requires additional power. The optoelectronic hybrid integration for future optical interface of chips not only requires co-package in form, but also co-designs to break the CEI barrier. By reconstructing and expanding the physical layer of OSI architecture, the optical engine that performs optoelectronic conversion can be incorporated into the framework of VLSI circuit co-design to realize the organic integration and full optoelectronic compatibility.

## CPO technology: applications, challenges, and China standardization progress^13^

### Status

**Fig. 23:**
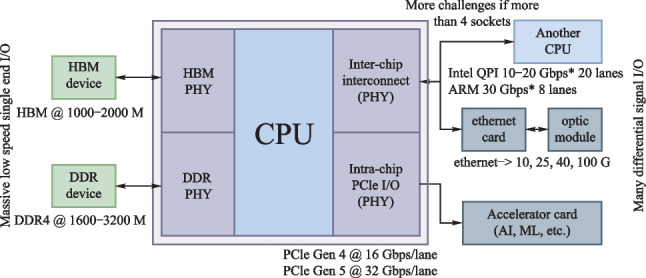
When CPU needs more I/O bandwidth but is limited by package, Optic I/O can help to solve the problem

Nowadays, the bandwidth requirement of switch ASIC and optical modules has reached up to 25.6 and 1.6 Tbps, respectively [80], raising power consumption to a degree that datacom equipment cannot tolerate in a system. Moreover, challenges in high-end server CPU design include package limitations and strict design rules, such as high-speed differential signal layout (as shown in Fig. 23). Furthermore, data-intensive computing tasks like AI demand much more data communication between AI chips [240], which requires the AI chip to be able to talk to another over the network interface.

Therefore, regarding the Switch/CPU/AI chip, there is an urgent need to implement a high-speed optical I/O port. Although optical technology is widely used in the telecom/datacom and datacenter operator market, it is still rare to see its application at chip-level and its integration with electronic chips.

Co-packaged optics (CPO), a recently-appeared new technology trend, replaces long reach SerDes with eXtreme Short Reach (XSR) SerDes to reduce the system’s power consumption, resulting in a very short distance between the electronic chip and optical transceiver chip. With that said, advanced packaging technology is necessary for integration between these chips.

Since CPO technology is still evolving, many technical challenges remain unsolved. Due to the fact that most optical technology currently used in the datacenter operator and telecom/datacom market are front-panel pluggable, a public-agreed standard also needs to be formed to establish a new economic system around CPO. OIF is now developing its CPO standard, and there is a collaboration between Facebook and Microsoft regarding CPO’s application in the datacenter operator.

In the rest of this section, we will describe the challenges we confronted in standardizing CPO technology and our efforts pushing forward to the standardization progress.

### Technology and market challenges

In CPO applications, the electronic and optical chips are placed very close to each other, raising issues including thermal, connector, laser source integration, etc. CPO also needs to compete with existing optical technology, such as front panel optical technology frequently used in datacom and telecom.

**Fig. 24:**
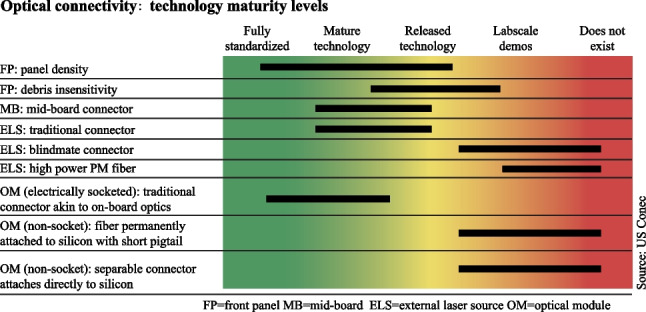
Technology maturity levels on optical connectivity [80]

Firstly, the connectivity is not mature (Fig. 24). To date, there are only two fully standardized technologies surrounding optic applications. One example is front-panel pluggable optics, which is very popular in datercenter operators. The other is on-board optics, which has the optical transceiver very close to the electronic chip on PCB but not at the same package substrate with the electronic chip. Connectivity regarding these two types of optic technology is more mature. In CPO application, optic fibers need to be attached to silicon with or without short pigtail, and external light source is needed to support blindmate mode. These technologies are neither mature nor currently available.

Secondly, it is challenging to integrate laser source with CPO module. The laser source should have features, such as high-temperature tolerance capability and small size. The quantum dot technology is a good candidate for laser sources from both temperature tolerance and size demand aspects. Japan AIO Core Corporation developed a tiny 100 Gbps optical transceiver with integrated quantum dot laser, TIA/Driver, and the rest components that support 100Gbps communication, whose size is only 5 mm × 5 mm [241]. Canada Ranovus Inc. developed a new-generation CPO sample based on multi-wavelength COMB laser, which uses quantum dot technology [246]. However, compared with quantum well technology, there are still many challenges, such as small modulation bandwidth and low output power. These challenges need to be solved before quantum dot technology can be used in mass production.

Thirdly, although using XSR SerDes instead of LR SerDes can reduce power consumption, the thermal problem remains a challenge in CPO applications. We conducted a simulation around a 16-CPO-module and 1 switch chip model. Results showed that the temperature of the central switch chip would be 151.76°C when running under a 5 m/s surface wind, which refers to a maximum specification acquired from the manual of a device designed to create cold wind. This means that the environment temperature is still too high for a chip to work even if it is cooled at maximum wind speed. Intel/Barefoot’s CPO module uses heat pipes to solve the thermal issue, which may be a feasible solution.

Fourthly, despite the fact that the CPO concept proposes a tighter integration between the electric and optical parts, how these parts are split is still not publicly agreed upon. Intel [141] integrates the SerDes, TIA/Driver, and optic parts into a photonic IC, while HP [242] integrates the payload IC, SerDes, and TIA/Driver into a single chip. ICT [243] chooses to integrate the payload IC, SerDes, and optical parts in a single chip by co-package technology, but with a standard interface between these parts.

Finally, a reduction in power alone may be insufficient to drive CPO adoption. Technology is not the only factor that impacts the success of CPO. The CPO solution should aim to achieve a compelling price reduction to replace front-panel pluggable optics. The optical engines should achieve an average selling price for 400G-DR4 optics from less than $1.20/Gbps in 2021 to less than $0.60/Gbps in 2024 [244]. As mentioned in Ref. [80], a 50% system‐level reduction in the cost per capacity compared with pluggable optics is feasible, arising from highly integrated many‐channel PICs with integrated waveguides, modulators, detectors, multiplexers, and V‐grooves for passively aligned fiber attachment.

### China standard progress

We founded the China Computer Interconnect Technology Alliance (CCITA) in 2020 to call for a group effort across the academy and industry to deal with these challenges and push the standardization and application of CPO technology forward. To date, CCITA includes 46 Chinese members covering the datacenter operator, server, chip design, optical module, connector, and other industries.

**Fig. 25:**
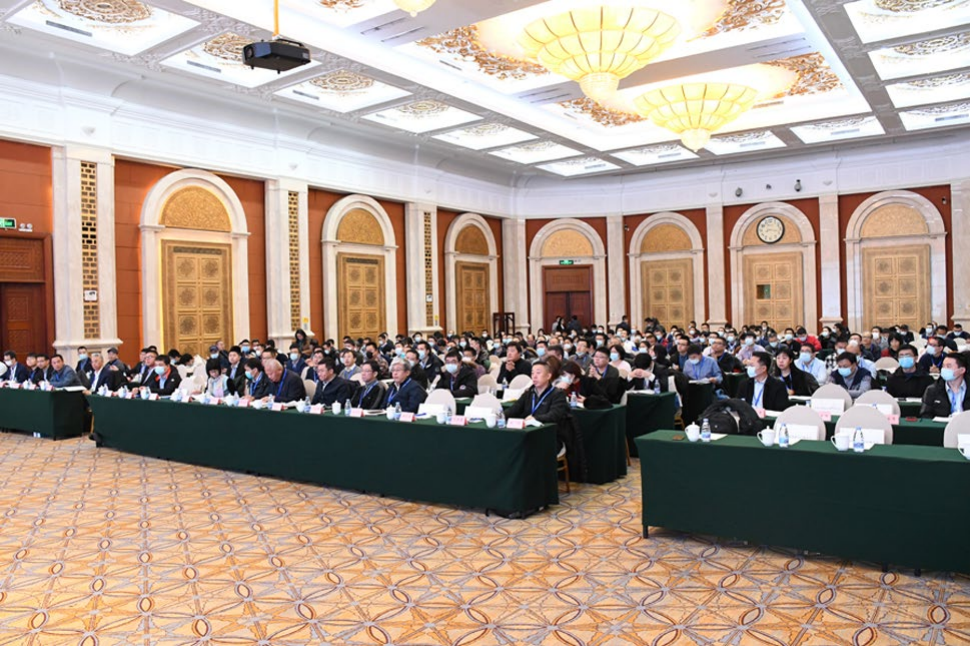
Scene of the 1st Conference of Chinese Interconnect Technology and Application

**Fig. 26:**
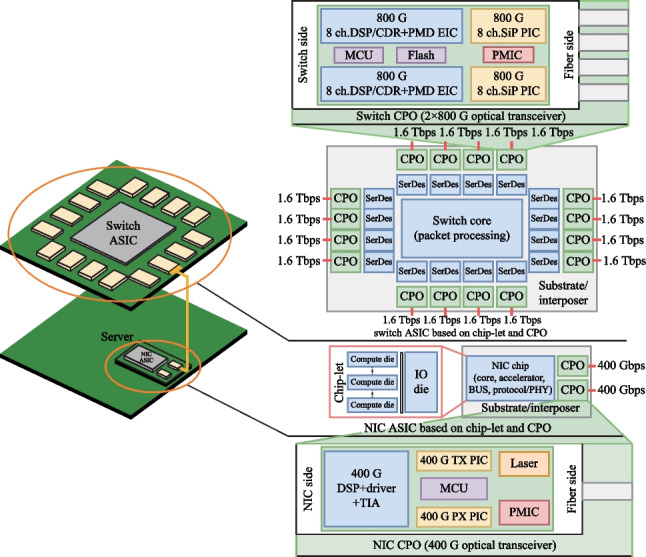
CCITA CPO and chiplet standard outline

In December 2020, CCITA took the lead and organized the 1st Conference of Chinese Interconnect Technology and Application with over 450 participants, as shown in Fig. 25. In May 2021, we charted two standard projects in the China Electronics Standardization Association, one surrounding CPO, and the other surrounding chiplet technology, which will facilitate the development of CPO, as shown in Fig. 26. There are 5 scenarios covered by these two standard projects. The CPO standard covers the CPO application in switches and network interface cards (NIC), while the chiplet standard covers chiplet scenarios, including Compute-to-Compute, Compute-to-I/O, and Compute-to-Others.

Different from any existing CPO standards, e.g., OIF’s CPO standard, our standard project aims to build a complete CPO interconnect chain from switches to NIC in server. Two reasons are listed below, including the fact that NIC is the unique feature of the China CPO standard. Firstly, the connector and CPO module must be of the same specification for applications in both switch and NIC sides, similar to AOC, a widely-used front-panel pluggable optical module standard in datacenter operator. Secondly, the NIC will soon confront the same power problem the switch has already met when the data rate per lane reaches up to 100Gbps, so it is necessary to consider both switch and NIC in the standard project. With that said, the benefit of the adoption of CPO on NIC side is to ensure that the switch and NIC have a uniform connectivity form factor in future applications, and the NIC side can also benefit from less power consumption.

An example of the China CPO standard application is shown in Fig. 26, which is the Switch-to-NIC scenario. A switch box is built via chiplet technology to connect its switch core and XSR SerDes. Then, it connects to 16 × 1.6Tbps CPO modules through XSR SerDes. A CPO module on the switch will be composed of a 2 × 800G optic transceiver chip along with other components, such as DSP and TIA/Driver. Between the switch box and NIC in the server, there is a break-out CPO optical cable at a 1:4 ratio. On the NIC side, the NIC chip connected to the 2 × 400Gbps CPO module is based on a 400Gbps optic transceiver. Ether SerDes on the switch side or NIC chip side are XSR, so switches and NIC chips can reach higher performance with acceptable power consumption. This is just one example of the China CPO standard application. Other applications could involve CPU to CPU in/between server and AI chip to AI chip in/between server.

The standard specification has just passed the proposal collective phase and will hopefully pass the draft phase by the end of this year. We will tape out a test chip next year to test the standard specification feasibility and release the standard at the end of next year.

### Concluding remark

Although CPO is a good technology for improving the performance of electronic chips, there are still many challenges to resolve before mass production and application. Meanwhile, technology is not the only factor that affects the success of CPO and increasing integration density to reduce its price can be a way to acquire market domination.

To call for a group effort across the academy and industry to deal with these challenges, we founded the CCITA in 2020, charted two standard projects around CPO in 2021, and drafted two specifications covering 5 scenarios of CPO and chiplet. In the future, we will tape out a prototype chip to test its feasibility and release the standard specification at the end of next year.

**Acknowledgements**

This work was partially supported by the National Key Research and Development Program of China (No. 2019YFB2203004).

## Footnotes

1 Min Tan^1,2^ and Jiang Xu^3,4,5^
^1^School of Optical and Electronic Information, Huazhong University of Science and Technology. ^2^Wuhan National Laboratory for Optoelectronics, Huazhong University of Science and Technology. ^3^Department of Electronic and Computer Engineering, The Hong Kong University of Science and Technology. ^4^HKUST Fok Ying Tung Research Institute. ^5^The Hong Kong University of Science and Technology (Guangzhou).

2 Siyang Liu and Junbo Feng Chongqing United Micro-Electronics Center (CUMEC).

3 Hua Zhang and Chaonan Yao Hisense Broadband Multimedia Technologies Co., Ltd.

4 Shixi Chen^1^ and Jiang Xu^1,2,3^ ^1^Department of Electronic and Computer Engineering, The Hong Kong University of Science and Technology. ^2^HKUST Fok Ying Tung Research Institute. ^3^The Hong Kong University of Science and Technology (Guangzhou).

5 Hangyu Guo, Gengshi Han, Zhanhao Wen, Bao Chen, Yu He, and Xuqiang Zheng Institute of Microelectronics, Chinese Academy of Sciences.

6 Ming Da^1^, Yaowen Tu^1^, and Min Tan^1,2^ ^1^School of Optical and Electronic Information, Huazhong University of Science and Technology. ^2^Wuhan National Laboratory for Optoelectronics, Huazhong University of Science and Technology.

7 Qiang Fu^1^, Min Tan^1,2^, and Nan Qi^3^ ^1^School of Optical and Electronic Information, Huazhong University of Science and Technology. ^2^Wuhan National Laboratory for Optoelectronics, Huazhong University of Science and Technology. ^3^State Key Laboratory of Superlattices and Microstructures, Institute of Semiconductors, Chinese Academy of Sciences.

8 Dan Li and Li Geng Faculty of Electronic and Information Engineering, Xi’an Jiaotong University.

9 Song Wen^1^, Haiyun Xue^1^, Fenghe Yang^2^, Huimin He^1^, and Fengman Liu^1^ ^1^Institute of Microelectronics, Chinese Academy of Sciences. ^2^Zhangjiang Laboratory.

10 Da Ming^1^, Yuhang Wang^1^, Ciyuan Qiu^2^, and Min Tan^1,3^ ^1^School of Optical and Electronic Information, Huazhong University of Science and Technology. ^2^The State Key Laboratory of Advanced Optical Communication Systems and Networks, Department of Electronic Engineering, Shanghai Jiao Tong University. ^3^Wuhan National Laboratory for Optoelectronics, Huazhong University of Science and Technology.

11 Guangcan Mi, Yanbo Li, and Tianhai Chang Huawei Technologies Co., Ltd.

12 Lai Mingche and Luo Zhang College of Computer, National University of Defense Technology.

13 Qinfen Hao and Mengyuan Qin Institute of Computing Technology, Chinese Academy of Sciences.
